# Physiological Responses to Swimming-Induced Exercise in the Adult Zebrafish Regenerating Heart

**DOI:** 10.3389/fphys.2018.01362

**Published:** 2018-10-01

**Authors:** Mireia Rovira, Daniel M. Borràs, Inês J. Marques, Carolina Puig, Josep V. Planas

**Affiliations:** ^1^Departament de Biologia Cellular, Fisiologia i Immunologia, Facultat de Biologia, Universitat de Barcelona, Barcelona, Spain; ^2^Research and Development Department, GenomeScan B.V., Leiden, Netherlands; ^3^Institute of Anatomy, University of Bern, Bern, Switzerland

**Keywords:** swimming, exercise, heart, regeneration, cardiomyocyte, proliferation, zebrafish

## Abstract

Exercise promotes a set of physiological responses known to provide long-term health benefits and it can play an important role in cardioprotection. In the present study, we examined cardiac responses to exercise training in the adult zebrafish and in the context of cardiac regeneration. We found that swimming-induced exercise increased cardiomyocyte proliferation and that this response was also found under regenerating conditions, when exercise was performed either prior to and after ventricular cryoinjury (CI). Exercise prior to CI resulted in a mild improvement in cardiac function and lesion recovery over the non-exercise condition. Transcriptomic profiling of regenerating ventricles in cryoinjured fish subjected to exercise identified genes possibly involved in the cardioprotective effects of exercise and that could represent potential targets for heart regeneration strategies. Taken together, our results suggest that exercise constitutes a physiological stimulus that may help promote cardiomyogenic mechanisms of the vertebrate heart through the induction of cardiomyocyte proliferation. The zebrafish exercise model may be useful for investigating the potential cardioprotective effects of exercise in teleost fish and to contribute to further identify and develop novel avenues in basic research to promote heart regeneration.

## Introduction

Regular exercise initiates cardiovascular and respiratory changes in order to supply and deliver oxygen and nutrients to the working muscles and other tissues. Apart from the acute cardiac responses that are put in place to meet the increased energetic demands, exercise training provides a set of physiological responses suggested to be cardioprotective on the heart or the cardiovascular system. Cardiac growth, improved myocardial contractility and increased coronary blood flow, reduced myocardial infarction in diseased hearts, cardiomyocyte renewal and, as recently reported, increased cardiomyogenesis, are some of the reported effects of exercise in the mammalian heart (Duncker and Bache, 2008; Lavie et al., 2015; Platt et al., 2015; Vujic et al., 2018). It is well-known that regular exercise is associated with reduced risk of mortality and prevalence of chronic diseases, especially those that compromise the cardiovascular system (Booth et al., 2012; Lee et al., 2012) and, therefore, there is a growing interest in elucidating the mechanisms driving exercise-induced cardioprotection in health and disease.

Among vertebrates, teleost fish hearts show a remarkable plasticity in response to environmental or physiological stressors, such as temperature, hypoxia, sexual maturation, diet, fish farming and exercise (Gamperl, 2004). In teleost fish, cardiovascular responses to exercise have been reported, such as changes in cardiac output, oxygen extraction efficiency, hematocrit, capillarization or cardiac growth (Davison, 1997; Gamperl, 2004; Jean et al., 2012). Similar to mammals, exercise-induced physiological responses in teleost fish may depend on the training intensity and duration and species analyzed, including fish species of commercial interest in view of the cardiac abnormalities reported in farmed fish (Poppe et al., 2009).

Among the fish species investigated, the zebrafish has received increased attention as a vertebrate model in the field of exercise physiology (Pelster et al., 2003; van der Meulen et al., 2006; LeMoine et al., 2010; Palstra et al., 2014; Rovira et al., 2017). Moreover, the zebrafish is currently a valuable complement to mammalian models in regenerative medicine. In contrast to mammals, adult zebrafish cardiomyocytes have the capacity to proliferate in response to a cardiac injury, contributing to the regeneration of the heart and its functional restoration (Foglia and Poss, 2016). While the mechanisms or factors involved in adult heart regeneration in zebrafish are being intensively studied, swimming tests have only been performed to evaluate the extent of cardiac injury on swimming performance (Wang et al., 2011; Gemberling et al., 2015) and the possible cardioprotective effects of exercise on heart regeneration in this species have not been investigated to date. Our group has previously demonstrated remarkable physiological responses in the adult zebrafish skeletal muscle under sustained aerobic exercise conditions (Palstra et al., 2014; Rovira et al., 2017) and, therefore, considering the reported plasticity of the teleost heart and specifically of the zebrafish heart, in this study we aimed to investigate the physiological effects of swimming-induced exercise in the healthy adult zebrafish heart as well as during regeneration after a cardiac injury. By subjecting adult zebrafish to the same swimming-induced exercise conditions previously established in our laboratory (Palstra et al., 2010), we found that exercise alone stimulates cardiomyocyte proliferation and that this response is maintained under cardiac injury. Furthermore, we identified genes that are induced or supressed in response to exercise in the lesioned heart that could contribute to the cardioprotective effects of exercise and that could represent potential targets for future cardiac regeneration studies. Our results suggest that exercise is a physiological stimulus that may induce cardioprotection in teleost fishes and contribute to the cardiac regenerative process in adult zebrafish through the induction of cardiomyocyte proliferation.

## Results

### Swimming-induced exercise effects on cell proliferation and cardiac function

To investigate the cardiac responses to swimming-induced exercise in adult zebrafish, we first examined whether swimming-induced exercise could lead to a hypertrophic cardiac response, as shown in mammals (Maillet et al., 2013) and other fish species (Davison, 1997; Jean et al., 2012). Morphometric analyses of the ventricular myocardium (Supplementary Figures S1A,B) revealed a significant increase in ventricular area when normalized by body weight (*P* = 0.026) in exercised zebrafish (Supplementary Figure S1H) but no significant changes were detected for other ventricular or cortical area measurements in response to swimming-induced exercise (Supplementary Figures S1C–G,I). Given that an increase in muscle mass in mammals is associated, at least in part, with increased protein synthesis (Goodman et al., 2011), we measured the activity of mTOR and the activity and phosphorylation status, respectively, of two downstream targets, p70S6K and 4E-BP1, in individual ventricles from exercised and non-exercised zebrafish. mTOR activity levels significantly increased in exercised over non-exercised fish (*P* = 0.017), but no significant changes were observed in p70S6K and 4E-BP1 activity or phosphorylation status, respectively (Supplementary Figures S1J,K). In addition, protein expression levels of myocyte enhancer factor 2 (Mef2), an essential transcription factor for cardiomyocyte differentiation (Hinits et al., 2012) that has been also linked to pathological hypertrophy (Kim et al., 2008), remained unchanged (Supplementary Figures S1J,K). We also examined the mRNA expression levels and localization of *nppa* and *postnb*, known markers for cardiac stress and fibrosis, respectively (González-Rosa et al., 2014; Ito et al., 2014), and no differences were found between exercised and non-exercised zebrafish indicating the absence of cardiac damage as a result of exercise training (Supplementary Figure S2). Therefore, our results suggest that the swimming-induced exercise training protocol applied did not result in a clear hypertrophic response nor in cardiac damage.

**Figure 1:**
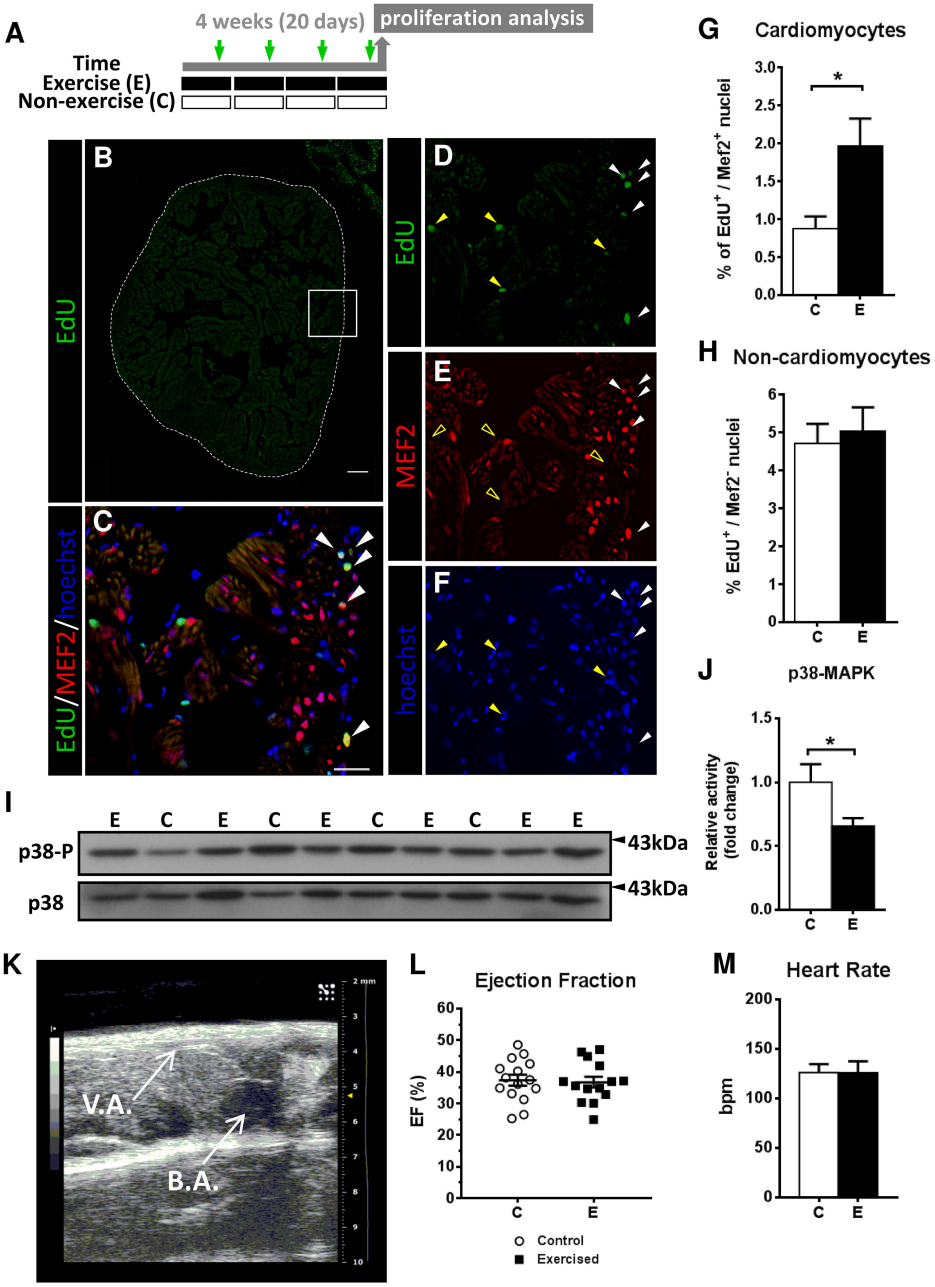
Evaluation of cardiomyocyte proliferation and cardiac function by 2D-echochardiography in response to swimming-induced exercise. **(A)** Schematic representation of the experimental design. Fish were induced to swim for a total of 20 days and injected with EdU (green arrows). **(B–F)** Representative section stained by EdU (green), Mef2c (red) and nuclei staining with Hoechst (blue). **(B)** Image of a whole ventricle section (dotted lines) labeled with EdU. Scale bar represents 100 μm. Higher magnification views of the framed area in **(B)**: **(C)** merged image from **(D–F)**. White arrowheads indicate EdU^+^-MEF2c^+^ cardiomyocyte nuclei. Yellow arrowheads indicate EdU^+^-MEF2c^−^ non-cardiomyocyte nuclei, as shown by the absence of MEF2c staining in (E) (open yellow arrowheads). Scale bar represents 10 μm. **(G)** There is a significant increase in the percentage of proliferative cardiomyocytes after exercise (*P* = 0.026). **(H)** Percentage of non-cardiomyocyte proliferative nuclei. Exercised (E, *n* = 6) and Control (C, *n* = 6) groups. **(I)** Representative SDS-PAGE of p38 MAPK as a cell differentiation/growth marker. **(J)** Quantification by densitometry of p38 MAPK activity as the ratio between phosphorylated p38 MAPK relative to total p38 MAPK (*P* = 0.020). Full-length blots are presented in Supplementary Figure S4. Data is expressed as fold change relative to the control group. Exercised (E, *n* = 12), Control (C, *n* = 6). **(K)** 2D echochardiography image of an adult zebrafish positioned ventrally (left side–caudal, right side–rostral) showing the ventricle apex (V.A.) and the bulbus arteriosus (B.A.). Atrium is not shown as is not easily visible. **(L)** Ejection fraction (%) parameter was measured to assess pumping ability of the ventricles. No significant differences were found. **(M)** Heart Rate (bpm, beats per min) was not altered between groups. Exercised (E, *n* = 13), Control (C, *n* = 15). *P < 0.05 (Mann-Whitney and Student's unpaired *t*-test). Bars represent the mean ± SEM.

Next, we investigated whether swimming-induced exercise caused a hyperplastic response in the adult zebrafish heart ventricle. We performed *in vivo* labeling with EdU followed by immunofluorescent detection of the cardiomyocyte-specific MEF2 protein to identify proliferating cardiomyocytes and measured the cardiomyocyte proliferation index (Figures 1A–F). Our results show a significant increase in the number of proliferating cardiomyocytes (EdU^+^/MEF2^+^) in ventricles from exercised over non-exercised zebrafish (*P* = 0.026; Figure 1G). In contrast, swimming did not affect the proliferation of non-cardiomyocyte cells (EdU^+^/MEF2^−^; Figure 1H). Consistent with the observed increase in cardiomyocyte proliferation, the activity of p38 mitogen activated protein kinase (p38-MAPK), an inducer of cell cycle arrest and promoter of differentiation that negatively regulates cardiomyocyte proliferation (Engel et al., 2005), was significantly decreased in exercised zebrafish ventricles (*P* = 0.02; Figures 1I,J). Analysis of protein expression levels in individual zebrafish ventricles of proliferating cell nuclear antigen (Pcna) and phospho-histone 3 (Ph3), two distinct proliferative cell cycle markers, revealed no differences between exercised and non-exercised fish (Supplementary Figure S3), likely because of the low proliferative index in the (uninjured) ventricles. Furthermore, the ventricular mRNA levels of neuroregulin1 (*nrg1*) and of two isoforms of the hypoxia-inducible factor 1 alpha (*hif1aa and hif1ab*), factors involved in cardiomyocyte proliferation in zebrafish (Jopling et al., 2012a; Gemberling et al., 2015), were not significantly altered in response to exercise (Supplementary Figure S2A). These results suggest that exercise may promote a small but significant level of cardiomyocyte proliferation in the adult zebrafish heart.

In order to investigate the potential functional consequences of swimming-induced exercise on cardiac function, we also measured ventricle ejection fraction (EF) and basal heart rate by 2D-echocardiography at the termination of the exercise training period (Figure 1K). However, no significant differences between exercised and non-exercised zebrafish were observed in EF (36.74 vs. 37.35%, respectively) or heart rate (126.1 and 125.8 bmps, respectively; Figures 1L,M).

Overall, these results indicate that swimming-induced exercise resulted in an increase in cardiomyocyte proliferation in the absence of significant changes in ventricular hypertrophy and cardiac function under the present exercise training conditions. In view of these results, we next investigated the effects of swimming-induced exercise on heart regeneration in response to cryoinjury using two experimental approaches: (1) exercise prior to ventricular cryoinjury and (2) exercise after ventricular cryoinjury.

### Effects of swimming-induced exercise prior to ventricular cryoinjury on heart regeneration

**Figure 2:**
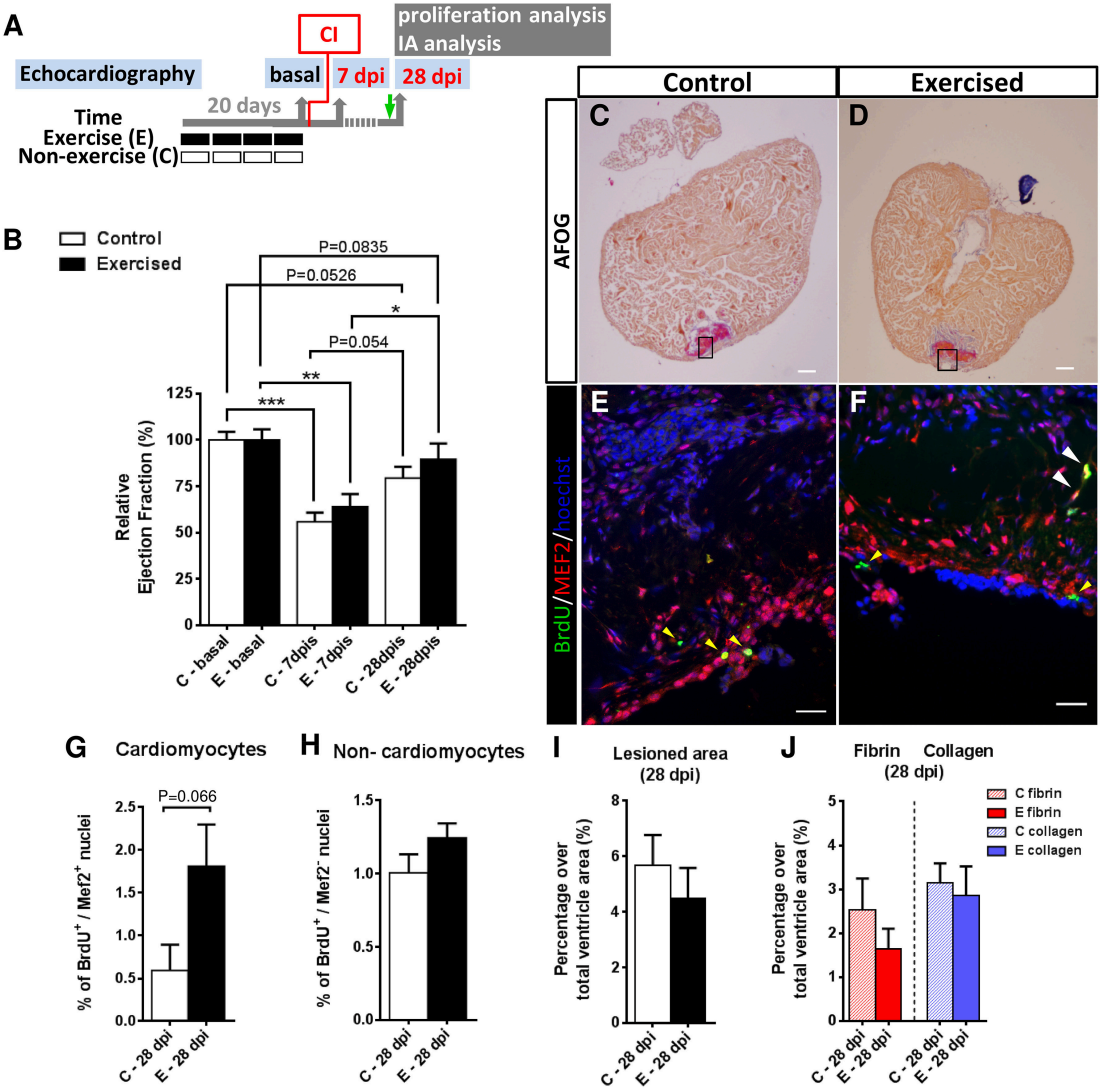
Effects of swimming-induced exercise prior to ventricular cryoinjury on heart regeneration. **(A)** Schematic representation of the experimental design. CI, cryoinjury; IA, injured area; BrdU injection (green arrow). **(B)** Evaluation of cardiac function after a cardiac injury in previously exercised (E) and non-exercised (C) fish. The relative change of ejection fraction shows a significant decrease at 7 days post injury (dpi; E, *P* = 0.0023; C, *P* = 0.0005) in comparison with basal conditions (non-injured ventricles) that is subsequently recovered after 28 dpi, with previously exercised fish (*P* = 0.0835) showing a better recovery (i.e., EF values at 28 dpi closer to basal values) than non-exercised fish (*P* = 0.0526). (E, *n* = 7; C, *n* = 7; *P < 0.05, **P < 0.01, ***P < 0.001, one-way ANOVA followed by Tukey's *post-hoc* test). **(C,D)** AFOG staining of representative ventricle sections from C and E fish at 28 dpi. Scale bar represents 100 μm. **(E,F)** Higher magnification views of serial sections corresponding to the framed areas in **(C,D)**. Immunofluorescence with BrdU (5-bromo-2′-deoxyuridine) combined with MEF2c. White arrowheads indicate BrdU^+^-MEF2c^+^ nuclei (cardiomyocytes). Yellow arrowheads indicate BrdU^+^-MEF2c^−^ nuclei (non-cardiomyocytes). Scale bar represents 10 μm. **(G)** Previously exercised fish show an increase in the percentage of proliferative cardiomyocyte nuclei at 28 dpi (*P* = 0.066, Mann-Whitney test) (E, *n* = 7; C, *n* = 7). **(H)** Percentage of proliferative non-cardiomyocyte nuclei. **(I)** After AFOG staining of ventricular sections from the same fish analyzed in B, the percentages of the total lesioned area in the ventricle at 28 dpi are shown (E, *n* = 7; C, *n* = 7). **(J)** From the same images analyzed in **(I)**, the percentages of fibrin and collagen over the total ventricle area were quantified. Bars represent the mean ± SEM.

We first investigated the influence of swimming-induced exercise prior to ventricular cryoinjury on cardiac function in a longitudinal study. After four weeks of swimming-induced exercise, we analyzed cardiac function by 2-D echocardiography in control and previously exercised animals at 0 (or basal, after exercise or resting), 7 and 28 days post-injury (dpi; Figure 2A). At 7 dpi, a significant decrease in ejection fraction (EF) in exercised (*P* = 0.0023) and non-exercised (*P* = 0.0005) fish was observed with respect to pre-injury (basal) values (Figure 2B), and although exercised fish showed slightly higher EF values than non-exercised fish when compared to their respective basal levels, the difference was not significant. At 28 dpi, EF values from exercised and non-exercised fish were not significantly different from pre-injury EF values, suggesting that cardiac function was restored in both groups, although again the difference with respect to basal levels was slightly lower in exercised (*P* = 0.0835) than in non-exercised fish (*P* = 0.0526) but without reaching statistical significance. Furthermore, exercised fish showed a significantly higher increase in EF (*P* = 0.029) between 7 and 28 dpi in contrast to non-exercised fish (*P* = 0.054; Figure 2B). Overall, these results suggest that swimming-induced exercise prior to ventricular cryoinjury may have resulted in a slight improvement in cardiac function during the process of regeneration.

We next investigated whether prior exercise training influenced cardiomyocyte proliferation during heart regeneration. We performed *in vivo* BrdU labeling of previously exercised and non-exercised zebrafish subjected to cryoinjury and examined cardiomyocyte proliferation at 28 dpi from the same individuals that had cardiac function assessed by 2D-echocardiography (Figures 2C–F). At the terminal sampling time of 28 dpi, we observed a 3-fold higher number of proliferating cardiomyocytes (BrdU^+^/MEF2^+^) in the injury-border zone of ventricles from exercised over non-exercised fish (*P* = 0.066), although the difference was not statistically significant (Figure 2G). In addition, the number of proliferating ventricular non-cardiomyocyte cells (BrdU^+^/MEF2^−^) was similar between exercised and non-exercised fish (*P* = 0.178; Figure 2H).

In order to quantify scar tissue clearance in the injured ventricular area we performed acid fuchsin orange G (AFOG) staining from the same individuals at 28 dpi (Figures 2C,D). Lesioned ventricular areas were smaller in exercised than in non-exercised zebrafish (4.5 and 5.7%, respectively), but no statistically significant differences were found between the two groups (Figure 2I). In order to analyze the extent of fibrosis in the regenerating ventricles, fibrin- and collagen-containing areas were quantified at 28 dpi. Fibrin (1.6% in exercised and 2.5% in non-exercised zebrafish) and collagen (2.8% in exercised and 3.14% in non-exercised zebrafish) ventricular areas were again slightly smaller in exercised than in non-exercised zebrafish, but no statistically significant differences between the two groups were found (Figure 2J). Overall, the increase in ventricular cardiomyocyte proliferation and the trend toward a slight cardiac functional recovery in previously exercised over non-exercised zebrafish, is not associated with a statistically significant reduction in the lesioned or fibrotic area, at least at 28 dpi.

### Effects of swimming-induced exercise after ventricular cryoinjury on heart regeneration

In view of the effects of exercise on cardiomyocyte proliferation in the absence of or prior to cardiac injury, we set out to investigate the effects of swimming-induced exercise after ventricular cryoinjury at the cellular and molecular levels. We first established that cryoinjured zebrafish were able to tolerate our swimming-induced exercise conditions at 3 dpi (data not shown) and examined possible functional differences in cryoinjured ventricles between exercised and non-exercised zebrafish by monitoring critical swimming speed (U_crit_; Supplementary Figure S4A). We subjected (untrained) cryoinjured zebrafish to resting conditions or to the swimming-induced exercise protocol for 5 consecutive days (from 3 to 7 dpi). After either swimming or resting during 5 consecutive days (from 3 to 7 dpi), no differences in U_crit_ average values between the two groups were found and these were similar to those of non-injured (non-exercised) zebrafish used as reference control (Supplementary Figure S4B). These results suggest that swimming-induced exercise during the first days of heart regeneration or a localized ventricular injury, such as cryoinjury, do not compromise adult zebrafish swimming performance. Interestingly, ventricular amputation in adult zebrafish does not compromise swimming performance either and it is only when cardiomyocyte depletion is distributed throughout the whole heart that swimming performance is reduced (Wang et al., 2011).

**Figure 3:**
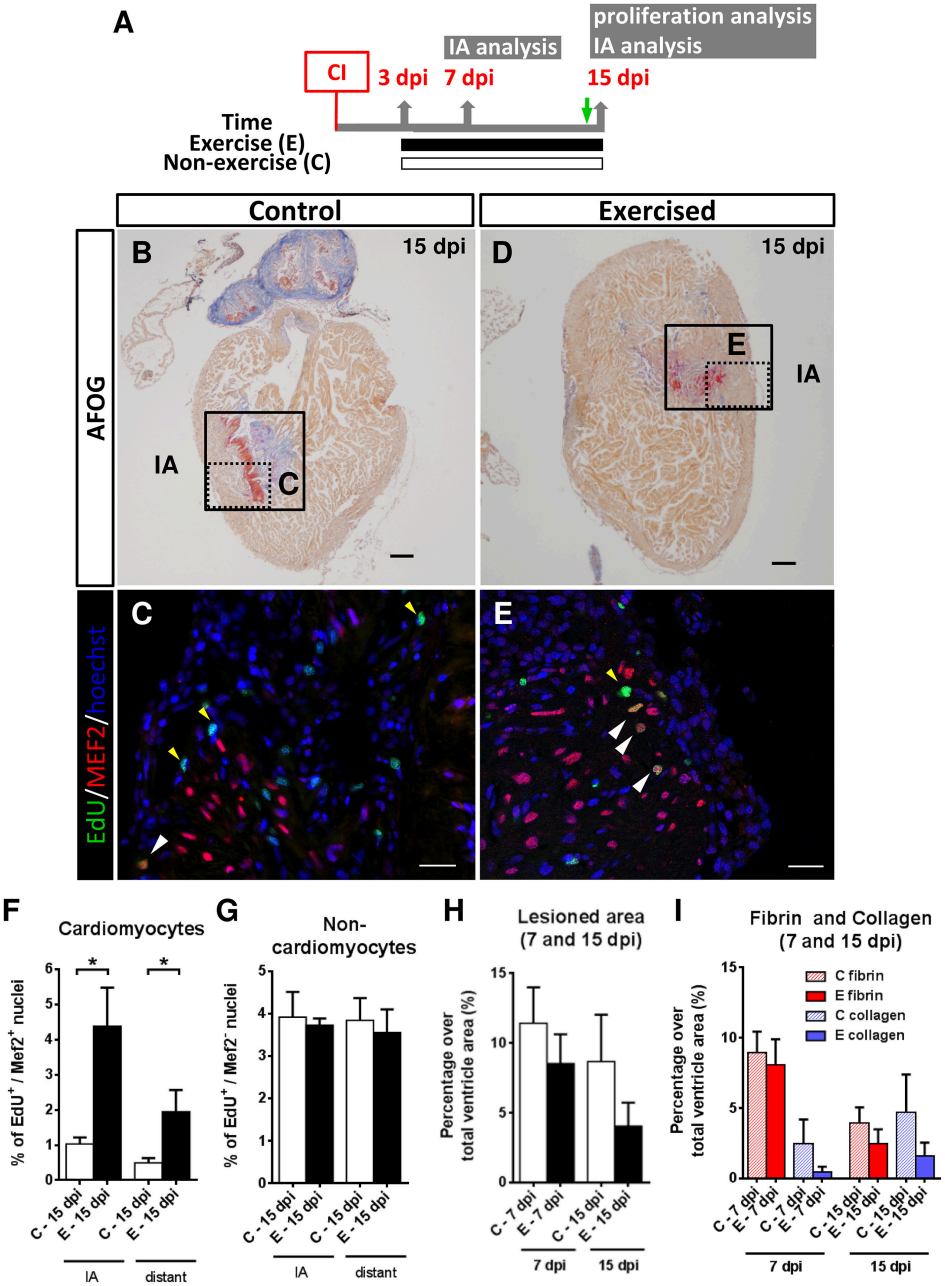
Effects of swimming-induced exercise after ventricular cryoinjury on heart regeneration. **(A)** Schematic representation of the experimental design. CI, cryoinjury; IA, injured area; EdU injection (green arrow). **(B–E)** After 13 days of swimming, lesioned hearts were examined for proliferation at 15 dpi. **(B,D)** AFOG staining. Boxed area shows the injured area (IA). Scale bars represent 100 μm. **(C,E)** Higher magnification views of the framed area in **(B,D)**. EdU labeling (green), MEF2 (red) and nuclei staining with Hoechst (blue). White arrowheads indicate EdU^+^-MEF2^+^ nuclei (cardiomyocytes). Yellow arrowheads indicate EdU^+^-MEF2^−^ nuclei (non-cardiomyocytes). Scale bar represents 10 μm. **(F–G)** Percentage of proliferative cardiomyocytes is increased both in the IA and distant to IA (*P* = 0.029 and *P* = 0.029, respectively; *P < 0.05, Mann Whitney test) **(F)**, whereas no differences were found for non-cardiomyocyte nuclei **(G)**. **(H,I)** Fibrotic area differences at 7 and 15 dpi after AFOG staining. **(H)** Total lesioned area. **(I)** Fibrin and collagen deposition quantification from the same images analyzed in I. 7 dpi: E, *n* = 5; C, *n* = 5 and 15 dpi: E, *n* = 4; C, *n* = 4. Bars represent the mean ± SEM. dpi, days post-injury.

Next, we subjected (untrained) cryoinjured zebrafish for 12 consecutive days (from 3 to 15 dpi) to resting conditions or to swimming-induced exercise and samples of cryoinjured hearts were taken at 7 and 15 dpi (Figure 3A; Supplementary Figure S5). Cardiomyocyte proliferation in response to swimming-induced exercise was only assessed at 15 dpi by EdU labeling (Figures 3B–E). Our results indicate that cardiomyocyte proliferation (EdU^+^/MEF2^+^) increased significantly (4-fold; *P* = 0.029) in exercised over non-exercised lesioned zebrafish hearts at 15 dpi, both in the injury-border zone as well as in an area distant from it (Figure 3F). In contrast, proliferation of non-cardiomyocyte cells showed no statistically significant differences between exercised and non-exercised zebrafish (Figure 3G). Next, we quantified the extent of the injured area at 7 and 15 dpi in AFOG-stained sections. In ventricles from exercised zebrafish, the damaged area was reduced by 25.3% at 7 dpi and 53.3% at 15 dpi when compared to non-exercised zebrafish, although no statistically significant differences were found (Figure 3H). In order to analyze the extent of fibrosis in the regenerating ventricle, fibrin- and collagen-containing areas were quantified (Figure 3I). At 7 dpi, lesioned exercised ventricles contained less fibrin than lesioned non-exercised ventricles (8.0 and 8.9%, respectively) or collagen (0.4 and 2.5%, respectively), although the differences between exercised and non-exercised fish were not statistically significant. Similar results were obtained when comparing lesioned exercised and non-exercised ventricles at 15 dpi, with 2.4 and 3.9% fibrin areas in exercised and non-exercised ventricles, respectively, and with 1.5 and 4.7% collagen areas in exercised and non-exercised ventricles, respectively. These results, although not statistically significant likely as a result of the modest sample size, suggest a trend toward a reduction of ventricular lesioned area or fibrosis in fish exercised after cryoinjury, similar to that observed in fish exercised prior to cryoinjury.

### Transcriptomic response of swimming-induced exercise after ventricular cryoinjury on heart regeneration

**Figure 4:**
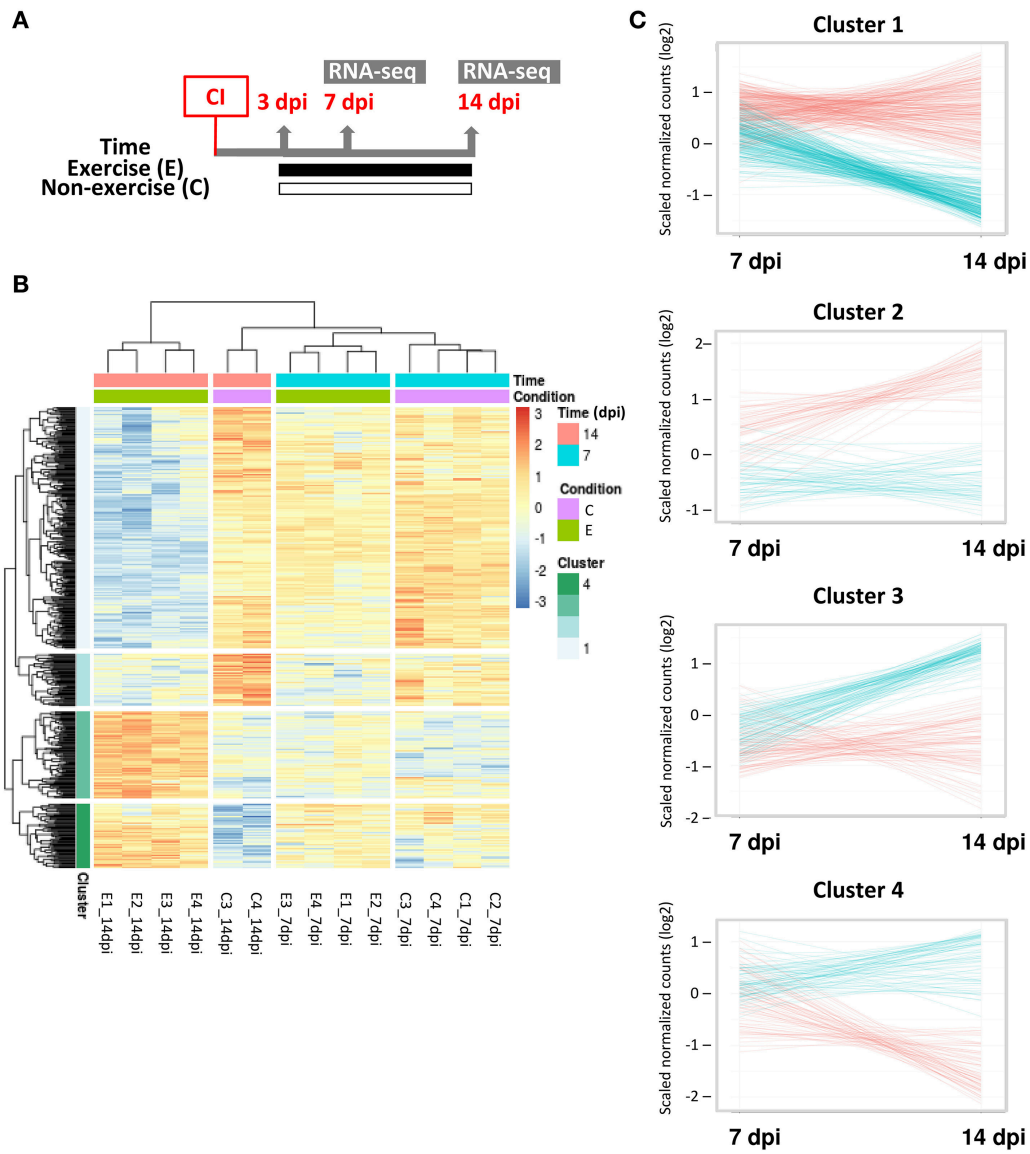
Transcriptomic analysis of adult zebrafish regenerating ventricles in response to swimming-induced exercise after ventricular cryoinjury. **(A)** Schematic representation of the experimental design. CI, cryoinjury; RNA-seq, RNA-sequencing. **(B)** Heat map for the 512 significant differential expressed genes (Padj < 0.1) from exercised (E) and control or non-exercised (C) zebrafish at two time points analyzed, 7 and 14 dpi. After hierarchical clustering, four clusters were obtained (see legend). **(C)** Expression trend of the genes included in each cluster. Gene counts (log2 scale) were plotted to show differential time expression between exercised (blue lines) and non-exercised (red lines) at 7 and 14 dpi.

We analyzed the transcriptomic changes in the adult zebrafish ventricle induced by exercise during the regenerative process from 7 to 14 dpi (Figure 4A) in order to gain insight on genes and gene networks involved in heart regeneration that are regulated in response to exercise. Sample clustering showed that samples are clustered by time (7 and 14 dpi) and condition (exercised or non-exercised; Supplementary Figure S6). Principal component analysis (PCA) showed that the distribution of the samples clearly represents the regenerative process over time, from left (7 dpi) to right (14 dpi) over the *x* axis (Supplementary Figure S6B). A total of 512 differentially expressed genes (DEGs; Padj < 0.1) were found, with 174 up-regulated and 338 down-regulated genes (Supplementary Table S1). Based on the gene expression data, hierarchical clustering grouped the samples by time (7 and 14 dpi) and condition (exercised or non-exercised) into four different clusters with distinct dynamic gene expression patterns, with 14 dpi being the time point with more relevant gene expression changes in response to exercise (Figure 4B). Cluster 1 and 2 comprised 278 and 60 DEGs, respectively, that were down-regulated by exercise from 7 to 14 dpi, whereas clusters 3 and 4 comprised 100 and 74 DEGs, respectively, that were up-regulated by exercise from 7 to 14 dpi (Figure 4C).

**Figure 5:**
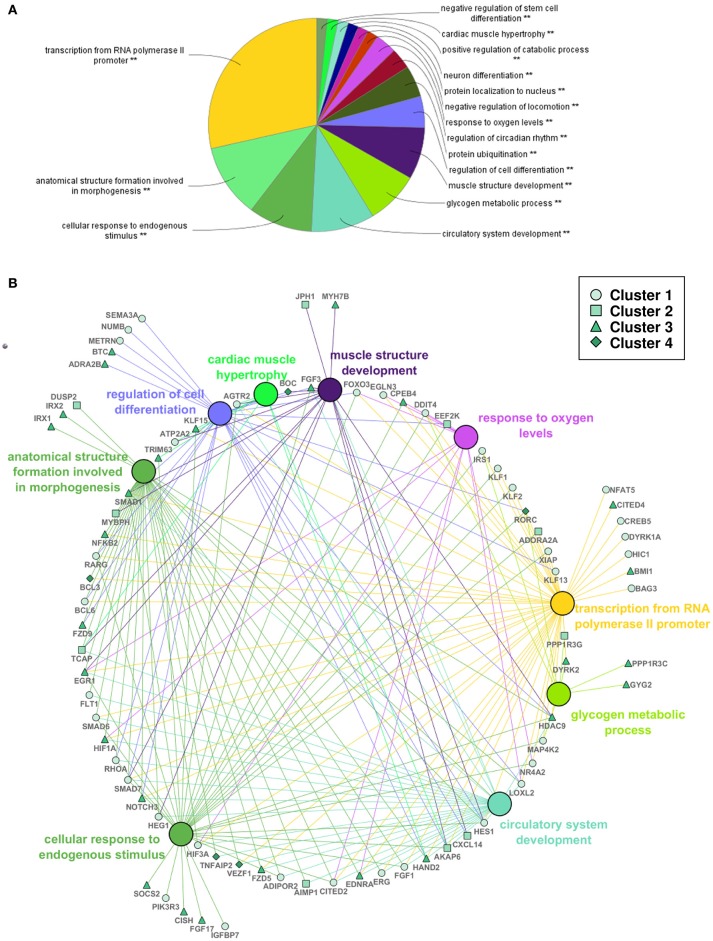
Functional analysis of differentially expressed genes in adult zebrafish regenerating ventricles in response to swimming-induced exercise. **(A)** Overview chart of the most significant GO terms (Biological Process) from all the significant DEGs (Padj < 0.1). **Indicates significant association of the genes represented in the GO terms (*P* < 0.05). **(B)** Selection of genes (human gene symbol) that were associated with GO terms in **(A)**. Large nodes show the GO term as shown in **(A)**. Small nodes represent selected genes associated with the GO terms (edge color) that correspond to the four clusters shown in Figure 4 (see legend).

A functional analysis of the complete set of DEGs was also performed to examine significantly enriched biological processes in response to swimming-induced exercise after ventricular cryoinjury (Figure 5A). Among others, exercise resulted in an enrichment of terms related to cellular and tissue structure and development, such as regulation of cell differentiation (e.g., *hdac9, numb, notch3, smad1, smad7*), muscle structure development (e.g., *hand2, ccnt2, ifrd1, jph1, myh7b*), cardiac muscle hypertrophy (e.g., *atp2a2, agtr2, tcap, klf15*), circulatory system development (e.g., *agtr2, aimp1, ednra, fgf1, fgf3, flt1, heg1, vezf1*) and anatomical structure formation involved in morphogenesis (e.g., *cited2, irx1, irx2, fzd5, fzd9*; Figure 5B). In addition, functional terms related to metabolism, such as glycogen metabolic process (e.g., *dryk2, gyg2, irs1, ppp1r3a*) and response to decreased oxygen levels [e.g., *hif1aa, hif1al (hif3a), egln3, loxl2, egr1*], suggestive of a hypoxic response, were also differentially regulated in exercised zebrafish (Figure 5B; Supplementary Table S2).

More specifically, factors related with adult zebrafish heart regeneration were differentially expressed including (a) members of the HIF-1α signaling pathway that positively regulate zebrafish heart regeneration and cardiomyocyte proliferation, such as *hif1aa* (up-regulated) and *hif1al* [down-regulated; (Jopling et al., 2012a)], (b) *hand2* (up-regulated), expressed during vertebrate cardiogenesis and also in the regenerating heart and that plays a role inducing cardiomyocyte proliferation (Lepilina et al., 2006; Schindler et al., 2014), (c) *nfkb2* (up-regulated), a subunit of the NF-kB transcription factor whose activity modulates cardiomyocyte proliferation and de-differentiation (Karra et al., 2015) and (d) *fgf17b* (up-regulated), suggested to be synthesized from the regenerating myocardium (including cardiomyocytes) in order to recruit epicardial cells and vascularize the new tissue (Lepilina et al., 2006; Supplementary Table S1). Exercise also induced the expression of genes involved in zebrafish fin regeneration, such as *klf17* (or *klf4b*) and *rarga* (Christen et al., 2010; Blum and Begemann, 2012), in limb regeneration in urodeles, such as *fgf1a* and *klf17* (or *klf4b*; Dungan et al., 2002; Maki et al., 2009), or in zebrafish hair cell regeneration, such as *klf17* and *midn* (Jiang et al., 2014). Other genes that have been related with cardiomyocyte proliferation include *p27 cdkn1bb, ccnd2* or *hdac9* (Yuan and Braun, 2017). From the significant differentially expressed genes, 60 unknown or non-described genes were found that could have a relevant role in the response of exercise in adult zebrafish heart regeneration.

## Discussion

Little is known about the beneficial and cardioprotective effects of exercise in teleost fish. In the present study, we investigated the cardiac response to swimming-induced exercise as well as its possible cardioprotective effects in adult zebrafish, an emerging experimental exercise model in vertebrates.

Here, we report for the first time in fish that exercise results in increased cardiomyocyte proliferation in the (uninjured) adult zebrafish heart. Our results provide support for previous studies that suggested that swimming may induce cardiomyocyte proliferation in the teleost heart (as determined by *pcna* mRNA expression levels or the number of cardiomyocytes or PCNA-positive cells; van der Meulen et al., 2006; Jean et al., 2012; Castro et al., 2013) and are in line with the known stimulatory effects of exercise on cardiomyocyte proliferation in the adult mammalian heart (Boström et al., 2010; Waring et al., 2014; Xiao et al., 2014; Vujic et al., 2018). Moreover, swimming-induced exercise in adult zebrafish also resulted in a decrease in the ventricular activity of p38-MAPK, a known inducer of cell cycle exit and differentiation in many cell types. p38α is a negative regulator of mammalian adult cardiomyocyte proliferation (Engel et al., 2005) and a similar mechanism in zebrafish cardiomyocytes has been found, whereby active p38α can block cardiomyocyte proliferation and heart regeneration (Jopling et al., 2012b). Therefore, our results support the notion that exercise may be a physiological stimulus to induce cardiac hyperplasia across vertebrates, a process that may contribute to the maintenance of tissue homeostasis in response to exercise by increasing cell turnover. Interestingly, despite the modest but significant cardiomyocyte proliferative effects of exercise in the healthy adult zebrafish heart, we did not observe a clear change in hypertrophy, except for the significant increase in ventricular area normalized by body weight and in mTOR activity, nor in cardiac function. The lack of a clear hypertrophic and functional response in the zebrafish heart under sustained swimming conditions was also reported in a previous study (Jean et al., 2012). Therefore, these results, combined with the lack of evidence for cardiac damage, suggest that sustained swimming conditions do not result in pathological cardiac stress in adult zebrafish. Further experiments should be performed in order to investigate conditions leading to a hypertrophic response in the adult zebrafish heart.

One of the objectives of the present study was to determine if swimming-induced exercise could exert subsequent cardioprotective effects in the context of cardiac injury. Studies investigating myocardial injury, ischemia or ischemia/reperfusion injury have provided evidence for a role of exercise in protecting the mammalian heart from subsequent myocardial damage, known as ischemic preconditioning (Maybaum et al., 1996; Domenech et al., 2002; Michaelides et al., 2003; Marongiu and Crisafulli, 2014). Interestingly, several studies have suggested that the fish heart can also be preconditioned under hypoxic and inflammatory conditions (Gamperl et al., 2001; Chen et al., 2005; de Preux Charles et al., 2016). On one hand, Gamperl et al. (2001) reported that after a prior anoxic exposure myocardial function was ameliorated from subsequent anoxia in the rainbow trout heart and Chen et al. (2005) reported that *in vitro* cardiac exposure to hypoxic conditions in goldfish, through activation of ATP-sensitive potassium ion channels, promoted the survival of ventricular myocytes exposed to hypoxic conditions. On the other hand, de Preux Charles et al. (2016) reported that thoracic surgery and a challenge with immunogenic agents prior to ventricular cryoinjury increased mitotic activity leading to the long-term remodeling of the adult zebrafish myocardium architecture. Moreover, long-term effects of exercise training in teleost fish have also been reported, lasting days or weeks (Young et al., 1994; Liu et al., 2009; Castro et al., 2011). Therefore, we hypothesize that by subjecting adult zebrafish to swimming-induced exercise for 4 weeks prior to cryoinjury, we may have induced a preconditioning-like effect as suggested by the increase in cardiomyocyte proliferation and the trend toward improved cardiac contractility, changes that were maintained for 28 days after training.

In the present study, we also investigated, for the first time in fish, the effects of swimming-induced exercise in the regenerating heart in adult zebrafish. Our results on the stimulation of cardiomyocyte proliferation by exercise during ventricular regeneration after cryoinjury provide further support for the proliferative capabilities of cardiomyocytes in the adult zebrafish heart and their response to swimming-induced exercise. Interestingly, the increased proliferation at the injury-border zone as well as in distant areas in exercised ventricles could suggest a higher contribution from the whole ventricle to the resolution of the injury in response to exercise in comparison with non-exercised regenerating hearts (Sallin et al., 2014).

In order to further understand the molecular mechanisms involved in the induction of cardiomyocyte proliferation during exercise on the regenerating adult zebrafish heart, we conducted transcriptomic profiling of the exercised regenerating ventricle. Our transcriptomic analyses of lesioned ventricles subjected or not to exercise led to the identification of a number of genes with regulated expression that have been related to regeneration and that could be involved in the observed exercise-induced cardiomyocyte proliferation. For example, we found that oxygen responsive genes were differentially regulated in the exercised regenerating zebrafish heart. Recently, hypoxia has been proposed to induce cardiomyocyte cell cycle re-entry in mammalian models (Puente et al., 2014; Kimura et al., 2015; Nakada et al., 2016). In the adult zebrafish heart, (chronic) hypoxia has shown to be mitogenic (Marques et al., 2008) especially after a cardiac injury, when the ensuing hypoxic response positively regulates heart regeneration through a mechanism involving HIF1α (Jopling et al., 2012a). Our results on the regulated expression of oxygen responsive genes, together with the enrichment of terms related with response to oxygen levels and the increase in cardiomyocyte proliferation, suggest a mechanism by which exercise may modulate the cardiac regeneration process through changes in metabolism, at least in the injured heart.

In addition, swimming-induced exercise also differentially regulated the expression of members of the Fibroblast Growth Factor family (FGF), such as *fgf17b* (Lepilina et al., 2006), *fgf3*, and *fgf1a*, found to be relevant in zebrafish cardiogenesis and neovascularization (Lepilina et al., 2006; Simoes et al., 2011). It is noteworthy that in mammals exercise promotes angiogenesis, regulation of the vascular tone and protection of coronary vasculature, changes that have been associated with the cardioprotective role of exercise in diseased hearts (Duncker and Bache, 2008; De Biase et al., 2014; Platt et al., 2015; Wang et al., 2015). Here, the enrichment of processes involved in the development of the circulatory system and, in particular, the differential regulation of pro-angiogenic genes from the FGF and HIF1α families, suggest a possible involvement of exercise to promote or control the vascular system during adult zebrafish heart regeneration. The possible angiogenic role of exercise in the zebrafish heart is supported by a previous study showing that under the same experimental conditions exercise promoted adult zebrafish skeletal muscle vascularization (Palstra et al., 2014) and by the observed coronary vessel regrowth in exercised and ablated fish (Farrell et al., 1990). Moreover, we identified other differentially expressed genes that can be associated with the observed exercise-induced cardiomyocyte proliferation in regenerating ventricles and that had not been previously characterized during zebrafish heart regeneration. For example, the transcription factor CITED4 was found to induce cardiomyocyte proliferation in the mammalian exercised heart (Boström et al., 2010). Here, *cited4b* was found to be significantly up-regulated in regenerating exercised ventricles at 15 dpi. Therefore, this transcription factor may represent a potential target for future cardiac regeneration studies. Taken together, in this study we identified a number of cardiac genes that are induced or suppressed in response to exercise in the lesioned heart that could contribute to the cardioprotective effects of exercise. In addition to intrinsic cardiac responses to exercise, it is also possible that the cardioprotective effects of exercise could be mediated by factors produced by the contractile skeletal muscle (i.e., myokines; Rovira et al., 2017), as suggested in mammals (Ouchi et al., 2016; Xi et al., 2016).

In conclusion, we found that swimming-induced exercise in adult zebrafish induced ventricular cardiomyocyte proliferation under normal and regenerating conditions. Under regenerating conditions, we identified an exercise-induced transcriptional program in the adult zebrafish heart, as well as a number of new potential key players and relevant biological processes. Our results highlight the potential of exercise as a physiological stimulus to contribute to the cardiac regenerative process and support the notion that the zebrafish is an excellent model to investigate yet unknown mechanisms of exercise-driven cardioprotection. Interestingly, important questions arise from our study, such as the possible contribution from the different cell types shaping the heart, the function of genes that have not been previously associated with heart regeneration that could potentially modulate the regenerative capacity of the zebrafish heart or the possibility that skeletal muscle- or cardiac-derived factors produced during exercise could have a cardioprotective effect as it has been recently demonstrated in mammals (Xi et al., 2016).

## Materials and methods

### Ethical approval

All animal procedures described herein have been approved by the Ethical Committee of the University of Barcelona under protocols DAAM7972 and 8969 to JVP and the Ethical Committee from the Comunidad Autonoma de Madrid. All experiments with live zebrafish were performed in accordance with relevant guidelines and regulations.

### Swimming-induced exercise training and experimental conditions

Exercise training conditions were performed as previously described (Palstra et al., 2010; Rovira et al., 2017). In brief, adult wild-type zebrafish of ~2.5 to 3 cm in body length (BL) were purchased from a local supplier. Exercised fish were housed in a 30 L swimming tunnel (Loligo Systems, Denmark) at the University of Barcelona while control fish were housed under the same density. Exercised adult zebrafish swam at their optimal swimming speed (i.e., speed at which the cost of transport is lowest; U_opt_) of 13 BL/s (0.4 m/s) for 6 h/day for 5 days/week in a total of 20 experimental days over a 4-weeks period (Palstra et al., 2010; Rovira et al., 2017) or in a total of 13 experimental days (see Experiments details below; Supplementary Methods; Supplementary Table S3; Supplementary Video S1).

### Exercise training and subsequent recovery from heart injury

After a four-week training period, exercised (*n* = 13) and non-exercised or control (*n* = 15) fish were transferred to the zebrafish facility of the Centro Nacional de Investigaciones Cardiovasculares (CNIC, Madrid) and acclimated for 2 days. Subsequently, fish were housed individually in 1.5 L tanks to perform a longitudinal study on heart lesion recovery after exercise. For that purpose, control and exercised fish were anesthetized by immersion in 0.168 mg/ml of buffered MS-222 and lesioned by ventricular cryoinjury as previously described (González-Rosa and Mercader, 2012) and kept individually in 1.5 L tanks for 28 days post-injury (dpi). At ~24 h (27 dpi) before being sacrificed, fish were anesthetized as indicated above and received an intraperitoneal injection of BrdU (5-bromo-2′-deoxyuridine; 30 μl at 2.5 mg/ml). Cardiac function in control (*n* = 7) and exercised fish (*n* = 7) was assessed by echocardiography, as previously described (González-Rosa et al., 2014), before the myocardial lesion (0 dpi), and at 7 and 28 dpi. Parameters measured were heart rate (bmp), systolic and diastolic area (mm^2^), systolic and diastolic volume (μl) and ejection fraction (%).

### Exercise training during recovery from heart injury

In order to evaluate the effects of exercise training during recovery from a heart lesion, non-exercised fish were subjected to heart cryoinjury as previously described (González-Rosa and Mercader, 2012) and allowed to recover for 48 h. At 3 dpi, a group of cryoinjured fish were introduced in the swimming tunnel and exercised daily under the conditions described above (13 BL/s for 6 h/day) continuously for a total of 13 experimental days, whereas a control group was maintained in resting conditions. At 7 and 14 dpi (after 5 and 12 experimental days, respectively), ventricle samples were obtained, flash frozen in liquid nitrogen and stored at −80°C. In order to assess *in vivo* cell proliferation, exercised fish and control fish were anesthetized at 14 dpi after the training session by immersion in 0.168 mg/ml of buffered MS-222 and received an intraperitoneal injection of EdU (1.25 mg/ml). Fish were sampled 24 h after the termination of exercise training (corresponding to 15 dpi) and heart samples were processed for histological procedures.

### Sampling

Fish were sampled after the experiments and euthanized by immersion in 0.250 mg/ml buffered MS-222. For the transcriptomic analysis, hearts were dissected, placed in sterile PBS to remove the atrium or bulbus arteriosus and the remaining ventricle was subsequently flash frozen in liquid nitrogen and stored at −80°C. Hearts were also collected for histology as previously described (González-Rosa and Mercader, 2012).

### RNA-sequencing and data analysis

Total RNA from three or four pooled ventricles from cryoinjured fish was isolated and homogenized in 150 μl of QIAzolLysis Reagent. Pooled samples at 7 (Exercised, *n* = 4; Control, *n* = 4) and 14 dpi (Exercised, *n* = 4; Control, *n* = 4) were processed with the miRNeasy MicroKit (Qiagen). The miRNeasy Micro Kit DNAse treatment was included for RNA-seq analysis to remove genomic DNA, following the manufacturer's specifications. Samples were sent to GenomeScan (Leiden, The Netherlands) for library preparation and sequencing. All samples passed the quality checks, being the lowest RQN = 8.9 (RNA Quality Number). Samples were sequenced using the next generation sequencing (NGS) platform IlluminaNextSeq 500 and the alignment was performed against the reference zebrafish genome GRCz10. Detected genes were considered differentially expressed if *P*-value (Benjamini-Hochberg) adjusted was <0.1 and any of the time points had a fold change >1.2. Human orthologs Ensembl Gene IDs were used to perform the Gene Onthology term enrichment analysis using ClueGO 2.3.2, a Cytoscape platform plugin (Bindea et al., 2009). Deposit of RNAseq data in the Gene Expression Omnibus (GEO) database at NCBI is under accession GSE100892.

### Histological processing and staining

Hearts stored in 70% ethanol were dehydrated, cleared in xylene and embedded in paraffin. Paraffin sections of 6 μm were cut using a Rotary 3003 microtome (Pfm Medical) and mounted on Superfrost slides (VWR). Hematoxylin and Eosin stained slides were scanned with NanoZoomer 2.ORS (Hamamatsu) and total and compact ventricle areas were quantified using the Tissuemorph software (Visiopharm). Acid Fuchsin Orange G (AFOG) staining was used to reveal fibrotic scar from cryoinjured heart samples (fibrin, red; collagen, blue; González-Rosa et al., 2014). Quantification of the injured area (fibrin and collagen) was calculated as a percentage over the total ventricular area and was averaged by all the lesioned sections for each heart. Image analysis was performed using ImageJ software (http://rsb.info.nih.gov/ij/).

### *In vivo* labeling of cardiomyocytes and immunohistochemistry

Twenty microliters of EdU (5-ethynyl-2′-deoxyuridine, Click-iT kit) at 1.25 mg/ml (Life Technologies) diluted in sterile PBS were injected in anesthetized fish (0.168 mg/ml of MS-222) intraperitoneally once a week and/or 24 h before being sacrificed, depending on the experiment. After the 20 experimental days, fish were euthanized and hearts were collected and processed for histological analyses. Briefly, 6 μm paraffin slides were dewaxed in xylene and rehydrated by a series of graded alcohols. After antigen retrieval was performed, slides were washed in 3% BSA/PBS, permeabilized in PBS-0.5% Triton for 20 min at room temperature and incubated with the Click-iT reaction cocktail in a wet chamber for 30 min at room temperature. After washing, blocking of non-specific binding was performed for at least 1 h in a wet chamber (5% BSA, 5% sheep serum/PBS). Immunofluorescence to specifically label cardiomyocyte cells was performed by using MEF2c (1:100, ab79436, Abcam) or MEF2 (1:50, sc-313, Santa Cruz) primary antibodies and AlexaFluor 555 (1:400, Life Technologies) secondary antibody, as previously reported (Jopling et al., 2012a; de Preux Charles et al., 2016; Hui et al., 2017). Nuclei staining was performed with Hoechst 33342 (1:2000, Thermo Scientific) and slides were mounted in ProLong Gold Antifade mounting media (Life Technologies). BrdU immunofluorescence in hearts at 28 dpi was performed as described above by using MEF2 antibody (1:50, Santa Cruz) and BrdU antibody (1:100, BD Biosciences) and anti-rabbit secondary antibody AlexaFluor 555 and anti-mouse secondary antibody Alexa Fluor 488 (1:400, Life Technologies), respectively. The cardiomyocyte proliferative index (proportion of EdU^+^ (or BrdU^+^)/Mef2^+^ nuclei over total number of Mef2^+^ nuclei) was calculated by averaging results from three non-consecutive separate sections in one slide (two slides in the case of 28 dpi hearts) containing the largest injury area per individual in the case of injured hearts or containing complete or near complete whole hearts per individual in the case of uninjured hearts. Similarly, quantification of proliferative non-cardiomyocyte cells was calculated by substracting the number of proliferative cardiomyocytes over the total cell number [EdU^+^ (or BrdU^+^) nuclei - EdU^+^ (or BrdU^+^)/Mef2^+^ nuclei over total number of Hoechst-stained nuclei]. Nuclei were counted manually using ImageJ software (http://rsb.info.nih.gov/ij/).

### Western blotting

Individual ventricles were lysed in RIPA buffer (Sigma) containing 1 mM phenylmethylsulfonyl fluoride (PMSF), 1X protease and phosphatase inhibitor cocktails (Sigma). The BCA kit (Thermo Scientific) was used for total protein quantification. A minimum of 20 μg of protein lysates were loaded in a SDS-PAGE gel and transferred to a PVDF membrane (Millipore). Membranes were blocked in 5% milk diluted in PBS/0.5% Tween for 1 h at room temperature and probed overnight at 4°C with the primary antibody. Membranes were washed in PBS/0.01% Triton and incubated for 2 h at room temperature with an anti-rabbit or anti-mouse HRP-conjugated secondary antibody (1:10,000, Jackson ImmunoResearch). The membranes were developed with the enhanced chemiluminescence method using Amersham ECL Prime Western Blotting Detection Reagent (GE Healthcare). Membranes were stripped with Restore Western Blot Stripping Buffer (ThermoScientific) following the manufacturer's indications to re-probe the membranes with the corresponding loading control. Effectiveness of the stripping procedure was checked every time by incubating the membrane with the secondary antibody. Primary antibodies used were: phospho-mTOR Ser2448 (1:100, Cell Signaling), mTOR (1:1000, Cell Signaling), phospho-p70 S6 Kinase Thr389 (1:200, Cell Signaling), p70 S6 Kinase (1:200, Cell Signaling), phospho-4E-BP1 Thr37/46 (1:500, Cell Signaling), 4E-BP1 (1:500, Cell Signaling), Mef2-pan (1:500, Anaspec), phospho-p38 Thr180/Tyr182 (1:200, Cell Signaling), p38 (1:100, Santa Cruz), and y-tubulin as a loading control (1:2000, Bethyl). Immunoreactive bands were visualized (LAS-3000; Fujifilm) and quantified with ImageJ software (http://rsb.info.nih.gov/ij/). Full-length blots are provided as Supplementary Material (Supplementary Figures S7, S8).

### Imaging

Fluorescent images were visualized by fluorescence microscopy using a Leitz DMIRB microscope and captured with a DFC360FX camera (Leica) or a Leica TCS SP2 confocal microscope (Leica Microsystems). White field images were obtained using a light microscope (Olympus) connected to a digital camera (DP70, Olympus).

### Statistical analysis

Statistical differences between two given groups were analyzed by two-tailed Student's *t*-test. Echocardiographic measurements were analyzed by one-way ANOVA followed by Tukey's honest significant test to control for multiple comparisons. When parametric assumptions of normality and equality of variance were not met, equivalent non-parametric tests were used as indicated in the figures. Results are expressed as mean ± standard error of the mean (SEM) and considered to be significant at *P* < 0.05. All statistical analyses were performed using GraphPad Prism6.

## Author contributions

MR and JP conceived and designed the experiments. MR performed the swimming experiments, histology, *in vivo* proliferation assays, heart lesions and lesion assessment, immunofluorescence, and protein and gene expression analyses; IM performed echocardiography, heart lesions and *in vivo* proliferation assays; CP performed protein expression analyses. MR and DB analyzed the data. MR and JP wrote the paper.

### Conflict of interest statement

The authors declare that the research was conducted in the absence of any commercial or financial relationships that could be construed as a potential conflict of interest.

## References

[B1] Bindea G. Mlecnik B. Hackl H. Charoentong P. Tosolini M. Kirilovsky A.. (2009). ClueGO: a cytoscape plug-in to decipher functionally grouped gene ontology and pathway annotation networks. Bioinformatics 25, 1091–1093. 10.1093/bioinformatics/btp101 19237447 PMC2666812

[B2] Blum N. Begemann G. (2012). Retinoic acid signaling controls the formation, proliferation and survival of the blastema during adult zebrafish fin regeneration. Development 139, 107–116. 10.1242/dev.065391 22096078

[B3] Booth F. W. Roberts C. K. Laye M. J. (2012). Lack of exercise is a major cause of chronic diseases. Compr. Physiol. 2, 1143–1211. 10.1002/cphy.c110025 23798298 PMC4241367

[B4] Boström P. Mann N. Wu J. Quintero P. A. Plovie E. R. Panáková D.. (2010). C/EBPβ controls exercise-induced cardiac growth and protects against pathological cardiac remodeling. Cell 143, 1072–1083. 10.1016/j.cell.2010.11.036 21183071 PMC3035164

[B5] Castro V. Grisdale-Helland B. Helland S. J. Kristensen T. Jørgensen S. M. Helgerud J.. (2011). Aerobic training stimulates growth and promotes disease resistance in Atlantic salmon (Salmo salar). Comp. Biochem. Physiol. Part A Mol. Integr. Physiol. 160, 278–290. 10.1016/j.cbpa.2011.06.013 21726657

[B6] Castro V. Grisdale-Helland B. Helland S. J. Torgersen J. Kristensen T. Claireaux G.. (2013). Cardiac molecular-acclimation mechanisms in response to swimming-induced exercise in Atlantic Salmon. PLoS ONE 8:e55056. 10.1371/journal.pone.0055056 23372811 PMC3555865

[B7] Chen J. Zhu J. X. Wilson I. Cameron J. S. (2005). Cardioprotective effects of K ATP channel activation during hypoxia in goldfish *Carassius auratus*. J. Exp. Biol. 208, 2765–2772. 10.1242/jeb.01704 16000545

[B8] Christen B. Robles V. Raya M. Paramonov I. Izpisúa Belmonte J. C. (2010). Regeneration and reprogramming compared. BMC Biol. 8:5. 10.1186/1741-7007-8-5 20089153 PMC2826312

[B9] Davison W. (1997). The effects of exercise training on teleost fish, a review of recent literature. Comp. Biochem. Physiol. Part A Physiol. 117, 67–75. 10.1016/S0300-9629(96)00284-8

[B10] De Biase C. De Rosa R. Luciano R. De Luca S. Capuano E. Trimarco B.. (2014). Effects of physical activity on endothelial progenitor cells (EPCs). Front. Physiol. 4:414. 10.3389/fphys.2013.00414 24550833 PMC3909827

[B11] de Preux Charles A. S. Bise T. Baier F. Sallin P. Jazwinska A. (2016). Preconditioning boosts regenerative programmes in the adult zebrafish heart. Open Biol. 6:160101. 10.1098/rsob.160101 27440423 PMC4967829

[B12] Domenech R. Macho P. Schwarze H. Sánchez G. (2002). Exercise induces early and late myocardial preconditioning in dogs. Cardiovasc. Res. 55, 561–566. 10.1016/S0008-6363(02)00334-6 12160953

[B13] Duncker D. J. Bache R. J. (2008). Regulation of coronary blood flow during exercise. Physiol. Rev. 88, 1009–1086. 10.1152/physrev.00045.2006 18626066

[B14] Dungan K. M. Wei T. Y. Nace J. D. Poulin M. L. Chiu I. M. Lang J. C.. (2002). Expression and biological effect of urodele fibroblast growth factor 1: relationship to limb regeneration. J. Exp. Zool. 292, 540–554. 10.1002/jez.10077 12115937

[B15] Engel F. B. Schebesta M. Duong M. T. Lu G. Ren S. Madwed J. B.. (2005). p38 MAP kinase inhibition enables proliferation of adult mammalian cardiomyocytes. Genes Dev. 19, 1175–1187. 10.1101/gad.1306705 15870258 PMC1132004

[B16] Farrell A. P. Johansen J. A. Steffensen J. F. Moyes C. D. West T. G. Suarez R. K. (1990). Effects of exercise training and coronary ablation on swimming performance, heart size, and cardiac enzymes in rainbow trout, *Oncorhynchus mykiss*. Can. J. Zool. 68, 1174–1179. 10.1139/z90-174

[B17] Foglia M. J. Poss K. D. (2016). Building and re-building the heart by cardiomyocyte proliferation. Development 143, 729–740. 10.1242/dev.132910 26932668 PMC4813344

[B18] Gamperl A. K. (2004). Cardiac plasticity in fishes: environmental influences and intraspecific differences. J. Exp. Biol. 207, 2539–2550. 10.1242/jeb.01057 15201287

[B19] Gamperl A. K. Todgham A. E. Parkhouse W. S. Dill R. Farrell A. P. (2001). Recovery of trout myocardial function following anoxia: preconditioning in a non-mammalian model. Am. J. Physiol. Regul. Integr. Comp. Physiol. 281, R1755–R1763. 10.1152/ajpregu.2001.281.6.R1755 11705758

[B20] Gemberling M. Karra R. Dickson A. L. Poss K. D. (2015). Nrg1 is an injury-induced cardiomyocyte mitogen for the endogenous heart regeneration program in zebrafish. Elife 4:e05871. 10.7554/eLife.05871 25830562 PMC4379493

[B21] González-Rosa J. M. Guzmán-Martínez G. Marques I. J. Sánchez-Iranzo H. Jiménez-Borreguero L. J. Mercader N. (2014). Use of echocardiography reveals reestablishment of ventricular pumping efficiency and partial ventricular wall motion recovery upon ventricular cryoinjury in the zebrafish. PLoS ONE 9:e115604. 10.1371/journal.pone.0115604 25532015 PMC4274112

[B22] González-Rosa J. M. Mercader N. (2012). Cryoinjury as a myocardial infarction model for the study of cardiac regeneration in the zebrafish. Nat. Protoc. 7, 782–788. 10.1038/nprot.2012.025 22461067

[B23] Goodman C. A. Mayhew D. L. Hornberger T. A. (2011). Recent progress toward understanding the molecular mechanisms that regulate skeletal muscle mass. Cell. Signal. 23, 1896–1906. 10.1016/j.cellsig.2011.07.013 21821120 PMC3744211

[B24] Hinits Y. Pan L. Walker C. Dowd J. Moens C. B. Hughes S. M. (2012). Zebrafish Mef2ca and Mef2cb are essential for both first and second heart field cardiomyocyte differentiation. Dev. Biol. 369, 199–210. 10.1016/j.ydbio.2012.06.019 22750409 PMC3927553

[B25] Hui S. P. Sheng D. Z. Sugimoto K. Gonzalez-Rajal A. Nakagawa S. Hesselson D.. (2017). Zebrafish regulatory T cells mediate organ-specific regenerative programs. Dev. Cell 43, 659.e5–672.e5. 10.1016/j.devcel.2017.11.010 29257949

[B26] Ito K. Morioka M. Kimura S. Tasaki M. Inohaya K. Kudo A. (2014). Differential reparative phenotypes between zebrafish and medaka after cardiac injury. Dev. Dyn. 243, 1106–1115. 10.1002/dvdy.24154 24947076

[B27] Jean M. J. Deverteuil P. Lopez N. H. Tapia J. D. Schoffstall B. (2012). Adult zebrafish hearts efficiently compensate for excessive forced overload cardiac stress with hyperplastic cardiomegaly. Biores. Open Access 1, 88–91. 10.1089/biores.2012.0201 23515072 PMC3559224

[B28] Jiang L. Romero-Carvajal A. Haug J. S. Seidel C. W. Piotrowski T. (2014). Gene-expression analysis of hair cell regeneration in the zebrafish lateral line. Proc. Natl. Acad. Sci. U.S.A. 111, E1383–E1392. 10.1073/pnas.1402898111 24706903 PMC3986165

[B29] Jopling C. Suñé G. Faucherre A. Fabregat C. Izpisua Belmonte J. C. (2012a). Hypoxia induces myocardial regeneration in zebrafish. Circulation 126, 3017–3027. 10.1161/CIRCULATIONAHA.112.107888 23151342

[B30] Jopling C. Suñè G. Morera C. Izpisua Belmote J. C. (2012b). p38α MAPK regulates myocardial regeneration in zebrafish. Cell Cycle 11, 1195–1201. 10.4161/cc.11.6.19637 22391208 PMC3679222

[B31] Karra R. Knecht A. K. Kikuchi K. Poss K. D. (2015). Myocardial NF-κB activation is essential for zebrafish heart regeneration. Proc. Natl. Acad. Sci. U.S.A. 112, 13255–13260. 10.1073/pnas.1511209112 26472034 PMC4629358

[B32] Kim Y. Phan D. Van Rooij E. Wang D. Z. McAnally J. Qi X.. (2008). The MEF2D transcription factor mediates stress-dependent cardiac remodeling in mice. J. Clin. Invest. 118, 124–132. 10.1172/JCI33255 18079970 PMC2129240

[B33] Kimura W. Xiao F. Canseco D. C. Muralidhar S. Thet S. Zhang H. M.. (2015). Hypoxia fate mapping identifies cycling cardiomyocytes in the adult heart. Nature 523, 226–230. 10.1038/nature14582 26098368

[B34] Lavie C. J. Arena R. Swift D. L. Johannsen N. M. Sui X. Lee D.. (2015). Exercise and the cardiovascular system. Circ. Res. 117, 207–219. 10.1161/CIRCRESAHA.117.305205 26139859 PMC4493772

[B35] Lee I.-M. Shiroma E. J. Lobelo F. Puska P. Blair S. N. Katzmarzyk P. T. (2012). Effect of physical inactivity on major non-communicable diseases worldwide: an analysis of burden of disease and life expectancy. Lancet 380, 219–229. 10.1016/S0140-6736(12)61031-9 22818936 PMC3645500

[B36] LeMoine C. M. Craig P. M. Dhekney K. Kim J. J. McClelland G. B. (2010). Temporal and spatial patterns of gene expression in skeletal muscles in response to swim training in adult zebrafish (*Danio rerio*). J. Comp. Physiol. B 180, 151–160. 10.1007/s00360-009-0398-5 19693513

[B37] Lepilina A. Coon A. N. Kikuchi K. Holdway J. E. Roberts R. W. Burns C. G.. (2006). A dynamic epicardial injury response supports progenitor cell activity during zebrafish heart regeneration. Cell 127, 607–619. 10.1016/j.cell.2006.08.052 17081981

[B38] Liu Y. Cao Z. D. Fu S. J. Peng J. L. Wang Y. X. (2009). The effect of exhaustive chasing training and detraining on swimming performance in juvenile darkbarbel catfish (*Peltebagrus vachelli*). J. Comp. Physiol. B 179, 847–855. 10.1007/s00360-009-0365-1 19462173

[B39] Maillet M. van Berlo J. H. Molkentin J. D. (2013). Molecular basis of physiological heart growth: fundamental concepts and new players. Nat. Rev. Mol. Cell Biol. 14, 38–48. 10.1038/nrm3495 23258295 PMC4416212

[B40] Maki N. Suetsugu-Maki R. Tarui H. Agata K. Del Rio-Tsonis K. Tsonis P. A. (2009). Expression of stem cell pluripotency factors during regeneration in newts. Dev. Dyn. 238, 1613–1616. 10.1002/dvdy.21959 19384853 PMC2749736

[B41] Marongiu E. Crisafulli A. (2014). Cardioprotection acquired through exercise: the role of ischemic preconditioning. Curr. Cardiol. Rev. 10, 336–348. 10.2174/1573403X10666140404110229 24720421 PMC4101198

[B42] Marques I. J. Leito J. T. Spaink H. P. Testerink J. Jaspers R. T. Witte F.. (2008). Transcriptome analysis of the response to chronic constant hypoxia in zebrafish hearts. J. Comp. Physiol. B 178, 77–92. 10.1007/s00360-007-0201-4 17828398 PMC2200676

[B43] Maybaum S. Ilan M. Mogilevsky J. Tzivoni D. (1996). Improvement in ischemic parameters during repeated exercise testing: a possible model for myocardial preconditioning. Am. J. Cardiol. 78, 1087–1091. 10.1016/S0002-9149(96)90057-0 8914868

[B44] Michaelides A. P. Andrikopoulos G. K. Oikonomou E. V. Psomadaki Z. D. Richter D. J. Dilaveris P. E.. (2003). Improved myocardial performance during repetitive exercise testing: the role of extracellular superoxide dismutase activity in a model of exercise-induced myocardial preconditioning. Am. Heart J. 146, 160–167. 10.1016/S0002-8703(03)00115-7 12851626

[B45] Nakada Y. Canseco D. C. Thet S. Abdisalaam S. Asaithamby A. Santos C. X.. (2016). Hypoxia induces heart regeneration in adult mice. Nature 541, 222–227. 10.1038/nature20173 27798600

[B46] Ouchi N. Ohashi K. Shibata R. Murohara T. (2016). Protective roles of adipocytokines and myokines in cardiovascular disease. Circ. J. 80, 2073–2080. 10.1253/circj.CJ-16-0663 27581346

[B47] Palstra A. P. Rovira M. Rizo-Roca D. Torrella J. R. Spaink H. P. Planas J. V. (2014). Swimming-induced exercise promotes hypertrophy and vascularization of fast skeletal muscle fibres and activation of myogenic and angiogenic transcriptional programs in adult zebrafish. BMC Genomics 15:1136. 10.1186/1471-2164-15-1136 25518849 PMC4378002

[B48] Palstra A. P. Tudorache C. Rovira M. Brittijn S. A. Burgerhout E. van den Thillart G. E.. (2010). Establishing Zebrafish as a novel exercise model: swimming economy, swimming-enhanced growth and muscle growth marker gene expression. PLoS ONE 5:e14483. 10.1371/journal.pone.0014483 21217817 PMC3013094

[B49] Pelster B. Sänger A. M. Siegele M. Schwerte T. (2003). Influence of swim training on cardiac activity, tissue capillarization, and mitochondrial density in muscle tissue of zebrafish larvae. Am. J. Physiol. Regul. Integr. Comp. Physiol. 285, R339–R347. 10.1152/ajpregu.00110.2003 12855415

[B50] Platt C. Houstis N. Rosenzweig A. (2015). Using exercise to measure and modify cardiac function. Cell Metab. 21, 227–236. 10.1016/j.cmet.2015.01.014 25651177 PMC4317572

[B51] Poppe T. T. Bornø G. Iversen L. Myklebust E. (2009). Idiopathic cardiac pathology in seawater-farmed rainbow trout, *Oncorhynchus mykiss* (Walbaum). J. Fish Dis. 32, 807–810. 10.1111/j.1365-2761.2009.01060.x 19531094

[B52] Puente B. N. Kimura W. Muralidhar S. A. Moon J. Amatruda J. F. Phelps K. L.. (2014). The oxygen-rich postnatal environment induces cardiomyocyte cell-cycle arrest through DNA damage response. Cell 157, 565–579. 10.1016/j.cell.2014.03.032 24766806 PMC4104514

[B53] Rovira i Berger M. (2016). Skeletal Muscle and Cardiac Adaptations to Swimming-Induced Exercise in Adult Zebrafish. Ph.D. thesis, University of Barcelona, Barcelona.

[B54] Rovira M. Arrey G. Planas J. V. (2017). Exercise-induced hypertrophic and oxidative signaling pathways and myokine expression in fast muscle of adult zebrafish. Front. Physiol. 8:1063. 10.3389/fphys.2017.01063 29326600 PMC5741866

[B55] Sallin P. de Preux Charles A. S. Duruz V. Pfefferli C. Jazwinska A. (2014). A dual epimorphic and compensatory mode of heart regeneration in zebrafish. Dev. Biol. 399, 27–40. 10.1016/j.ydbio.2014.12.002 25557620

[B56] Schindler Y. L. Garske K. M. Wang J. Firulli B. A. Firulli A. B. Poss K. D.. (2014). Hand2 elevates cardiomyocyte production during zebrafish heart development and regeneration. Development 141, 3112–3122. 10.1242/dev.106336 25038045 PMC4197543

[B57] Simoes F. C. Peterkin T. Patient R. (2011). Fgf differentially controls cross-antagonism between cardiac and haemangioblast regulators. Development 138, 3235–3245. 10.1242/dev.059634 21750034 PMC3133915

[B58] van der Meulen T. Schipper H. van den Boogaart J. G. Huising M. O. Kranenbarg S. van Leeuwen J. L. (2006). Endurance exercise differentially stimulates heart and axial muscle development in zebrafish (*Danio rerio*). Am. J. Physiol. Regul. Integr. Comp. Physiol. 291, R1040–R1048. 10.1152/ajpregu.00116.2006 16966387

[B59] Vujic A. Lerchenmüller C. Wu T. D. Guillermier C. Rabolli C. P. Gonzalez E.. (2018). Exercise induces new cardiomyocyte generation in the adult mammalian heart. Nat. Commun. 9:1659. 10.1038/s41467-018-04083-1 29695718 PMC5916892

[B60] Wang J. Panakova D. Kikuchi K. Holdway J. E. Gemberling M. Burris J. S.. (2011). The regenerative capacity of zebrafish reverses cardiac failure caused by genetic cardiomyocyte depletion. Development 138, 3421–3430. 10.1242/dev.068601 21752928 PMC3143562

[B61] Wang Y. Li M. Dong F. Zhang J. Zhang F. (2015). Physical exercise-induced protection on ischemic cardiovascular and cerebrovascular diseases. Int. J. Clin. Exp. Med. 8, 19859–19866. 26884896 PMC4723741

[B62] Waring C. D. Vicinanza C. Papalamprou A. Smith A. J. Purushothaman S. Goldspink D. F.. (2014). The adult heart responds to increased workload with physiologic hypertrophy, cardiac stem cell activation, and new myocyte formation. Eur. Heart J. 35, 2722–2731. 10.1093/eurheartj/ehs338 23100284 PMC4196078

[B63] Xi Y. Gong D.-W. Tian Z. (2016). FSTL1 as a potential mediator of exercise-induced cardioprotection in post-Myocardial Infarction Rats. Sci. Rep. 6:32424. 10.1038/srep32424 27561749 PMC5000295

[B64] Xiao J. Xu T. Li J. Lv D. Chen P. Zhou Q.. (2014). Exercise-induced physiological hypertrophy initiates activation of cardiac progenitor cells. Int. J. Clin. Exp. Pathol. 7, 663–9. 24551287 PMC3925911

[B65] Young P. S. Cech J. J Jr (1994). Optimum exercise conditioning velocity for growth, muscular development, and swimming performance in young-of-the-year striped bass (*Morone saxatilis*). Can. J. Fish. Aquat. Sci. 51, 1519–1527. 10.1139/f94-151

[B66] Yuan X. Braun T. (2017). Multimodal regulation of cardiac myocyte proliferation. Circ. Res. 121, 293–309. 10.1161/CIRCRESAHA.117.308428 28729454

