# Supplementary material for: Physiological Responses to Swimming-Induced Exercise in the Adult Zebrafish Regenerating Heart
Source: Front Physiol. 2018 Oct 1;9:1362. doi: 10.3389/fphys.2018.01362 (PMC6174316; doi:10.3389/fphys.2018.01362)
Supplement: Supplementary file 3 [file Table_3.docx]

| **Table S3. Primer sequences used for quantitative-real time PCR (qPCR).** | | | |
| --- | --- | --- | --- |
| **Gene** | **Sequence** | **Accession number** | **Amplicon (bp)** |
| ***rps15*** | F TTCAGGAAATTCACCTACAGAGG  R CCTCTGCCTGGCGCTATAC | NM_001001819 | 93 |
| ***rps18*** | F AGATGGGAAATACAGCCAGGT  R CCCCAGAAGTGACGGAGA | NM_173234 | 111 |
| ***rpl11*** | F CTGTTCGAGGAGCCAAGG  R TTGTTCTTTCTCAACTCGTACTCG | NM_001002139 | 76 |
| ***bactin*** | F GATCTTCACTCCCCTTGTTCA  R ATACCGGAGCCGTTGTCA | NM_131031 | 91 |
| ***hif1aa*** | F GGACGGGTTTCTTTTGGTTC  R CACTGTGACCAGTGAGTTCAATC | ENSDART00000044282 | 107 |
| ***hif1ab*** | F TGCAGTGCACCTGTCTCG  R TGGAAGGCTAACATCTTTGGA | ENSDART00000018500 |  |
| ***nppa*** | F AGGACTGCTGCTCCTGGTT  R GGCCTCCTCAAACTGCTG | NM_198800 | 118 |
| ***nrg1*** | F ACCACAGCAAACCTATTCATCA  R TCCTTCTCGCTCTCATTGC | NM_001044911 | 98 |
| ***postnb*** | F GCGTTACCAAGGTCACTCGT  R CCTCACCGAAATCTTCAATGACT | ENSDART00000017098 | 149 |
| ***tgfb1a*** | F CAGAAGATGAAGAAACCTTGGAG  R TGTCTAGCCCTGATATAGTGAAGAG | ENSDART00000060839 | 98 |
| Forward (F) and reverse (R) primer sequences, GeneBank (NM_) or Ensmbl (ENSDART) accession numbers and amplicon length are shown. | | | |
