# Supplementary material for: Physiological Responses to Swimming-Induced Exercise in the Adult Zebrafish Regenerating Heart
Source: Front Physiol. 2018 Oct 1;9:1362. doi: 10.3389/fphys.2018.01362 (PMC6174316; doi:10.3389/fphys.2018.01362)
Supplement: Supplementary file 5 [file Data_Sheet_1.PDF]

Rovira M. *et al.* (2018) – Physiological responses to swimming-induced exercise in the adult zebrafish regenerating heart

SUPPLEMENTARY FIGURES

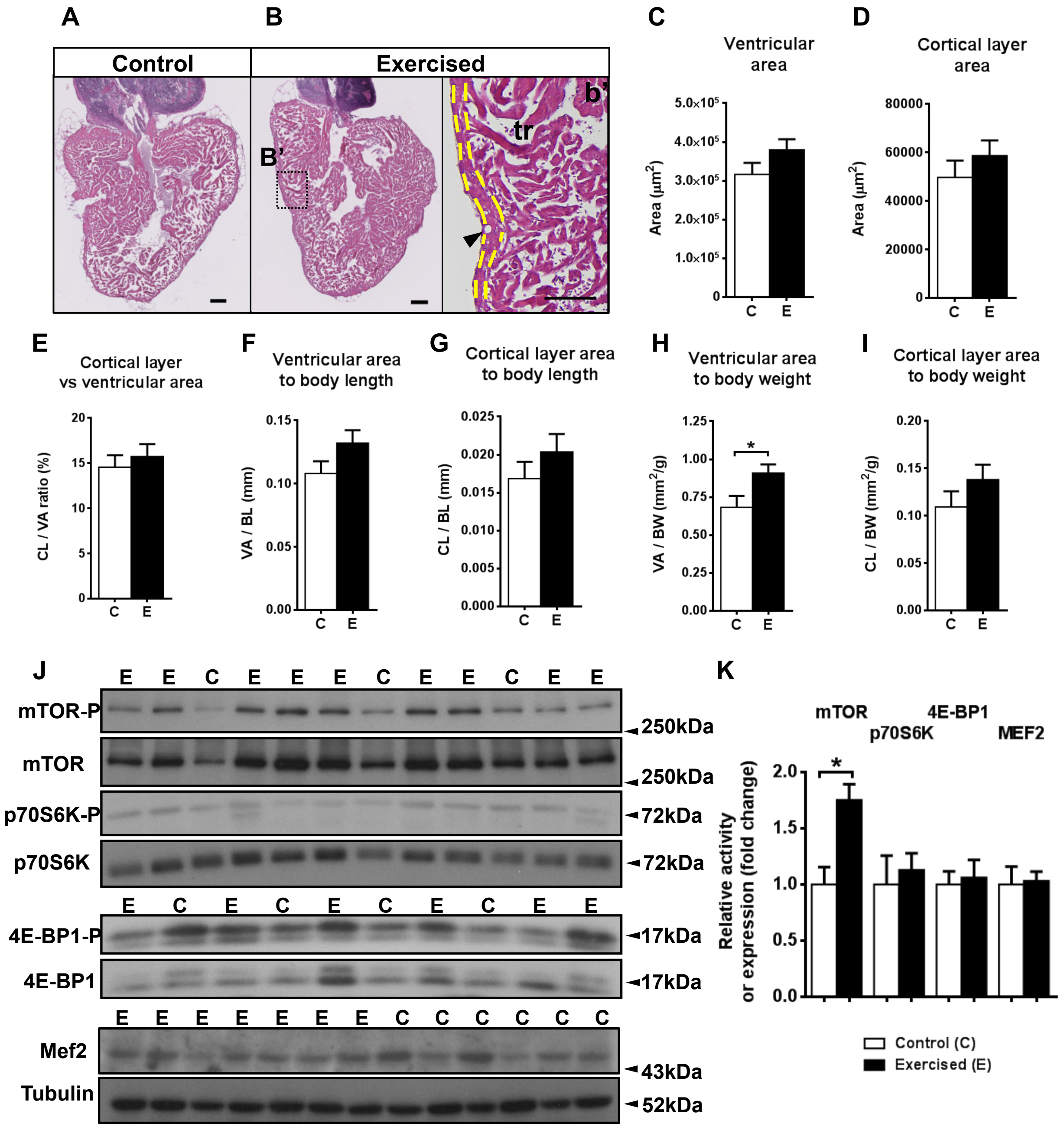

Supplementary Figure S1. Ventricular hypertrophic responses to swimming-induced exercise in healthy adult zebrafish. (A-B) Representative hematoxylin- and eosin-stained sections from control and exercised hearts used for quantification. **(B')** Zoomed area boxed in b. Yellow lines delineate the cortical myocardium. Arrowhead points to a coronary vessel in the ventricular wall. tr, trabecular myocardium. Scale bars represent 100 μm. **(C)** Quantification of the ventricular area (VA). **(D)** Quantification of cortical layer area (CL). **(E)** Ratio of CL/VA. (C, n=6, E, n=6; 12-20 sections per heart). **(F-G)** Ratio of VA and CL to body length (BL). **(H-I)** Ratio of VA and CL to body weight (BW). **(J)** Representative SDS-PAGE of mTOR pathway represented by mTOR, its downstream kinase p70S6K (E, n=9; C, n=3), the translation repressor protein 4E-BP1 (E, n=12; C, n=6) and the MEF2 transcription factor (E, n=7; C, n=6). Arrowheads indicate the molecular weight marker. **(K)** Quantification by densitometry of Western blots. Activity levels of mTOR (P=0.017) and p70S6K and phosphorylation status of 4E-BP1 represent the levels of the phosphorylated form (P) relative to the total form. MEF2 protein expression levels are shown relative to tubulin. Full-length blots are presented in Supplementary Figure S7. Data are expressed as fold change relative to the control group. *P<0.05 (Mann-Whitney test). Exercised (E) and Control (C) group. Bars represent the mean ± SEM.

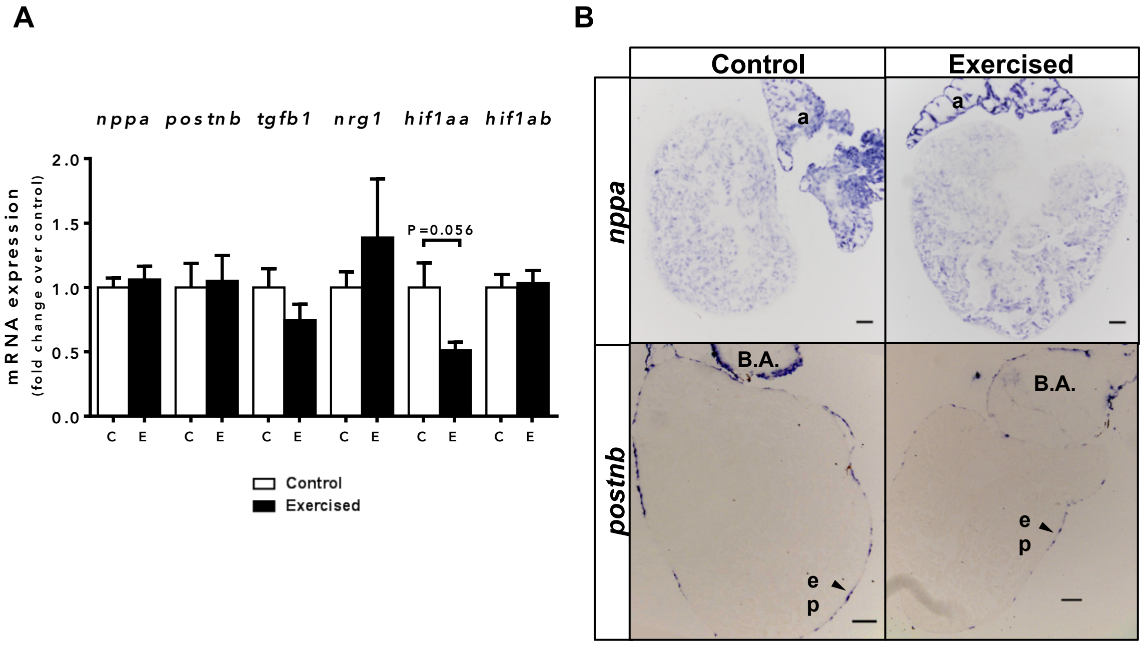

Supplementary Figure S2. Cardiac marker gene expression in exercised adult zebrafish. (A) mRNA expression levels of *nppa (*natriuretic peptide A), a marker for cardiac stress; *postnb* (periostin-b), a fibroblast marker expressed after cardiac injury; *tgfb1* (transforming growth factor beta 1), a growth factor that has also been related with fibrosis; *nrg1* (neuroregulin), a growth factor known to induce cardiomyocyte hyperplasia; and *hif1aa* and *hif1ab* (hypoxia-inducible factor 1 alpha), also known to induce cardiomyocyte proliferation, in exercised (E) over non-exercised (C) adult zebrafish pooled ventricles (n=2) after 4 weeks (20 days) of training. Data are expressed as fold induction above the control group, which was set to 1. Bars represent the mean ± SEM (E, n=5; C, n=5). **(B)** In situ hybridization of *nppa* and *postnb* in hearts from exercised and non-exercised zebrafish. No differences in mRNA localization were observed between the groups. Note that *nppa* is usually highly expressed in the atrium (a) (González-Rosa JM, et al. 2014) and that *postnb* has a weak expression in adult healthy hearts in the epicardium (ep, arrowheads) and bulbus arteriosus (B.A.) (Ito et al. 2014). Scale bars represent 100 µm. Descriptions of the methods used for quantitative real time PCR and in situ hybridization can be found in Supplementary Methods.

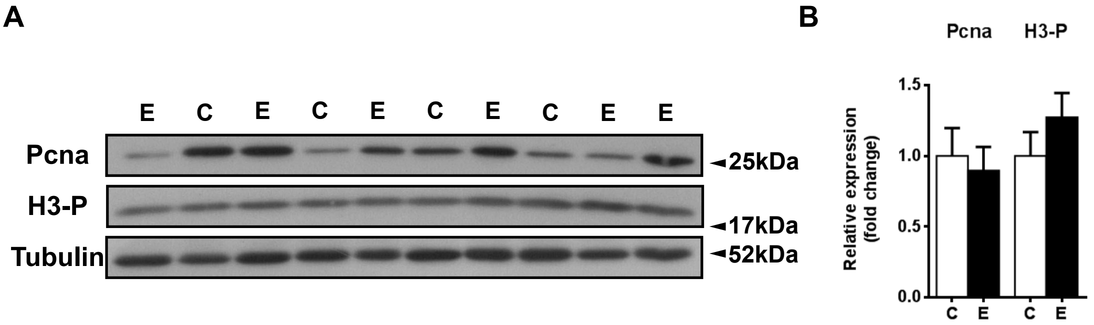

Supplementary Figure S3. Protein expression levels of cell proliferation markers in individual ventricles in response to swimming-induced exercise. (A) Representative SDS-PAGE of cell proliferation markers proliferating cell nuclear antigen (Pcna) and phosphorylated histone 3 (H3-P) (E, n=12; C, n=6). Arrowheads indicate the molecular weight marker. **(B)** Quantification by densitometry of Western blots shown in (a). Protein expression levels of Pcna and H3-P were analyzed relative to tubulin. Full-length blots are presented in Supplementary Figure S8. Data are expressed as fold change relative to the control group. Exercised (E) and Control (C) group. Bars represent the mean ± SEM.

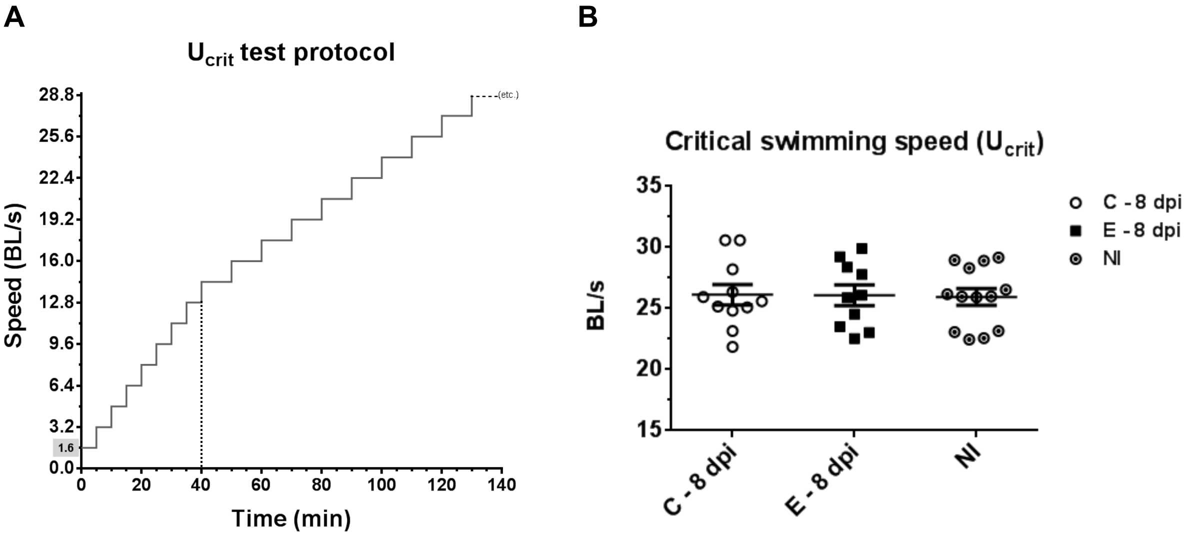

Supplementary Figure S4. Critical swimming performance (U_{crit}) test at 8 dpi in exercised and non-exercised zebrafish. (A) Ramp-U_{crit} test protocol. Speed increment was of 1.6 BL/s for each step. Vertical dotted line indicates the end of the first seven initial 5 min step increments and the beginning of the following 10 min step increments that continued until fish fatigued. **(B)** Critical swimming speed of non-exercised (C), exercised (E) and non-injured (NI) zebrafish, that were included as reference. No statistically significant differences were found (one-way ANOVA followed by Tukey's post-hoc test). E, n=10; C, n=11; NI, n=13. Bars represent the mean ± SEM. BL/s, body lengths per second; dpi, days post-injury. Descriptions of the methods used for determining the critical swimming speed can be found in Supplementary Methods.

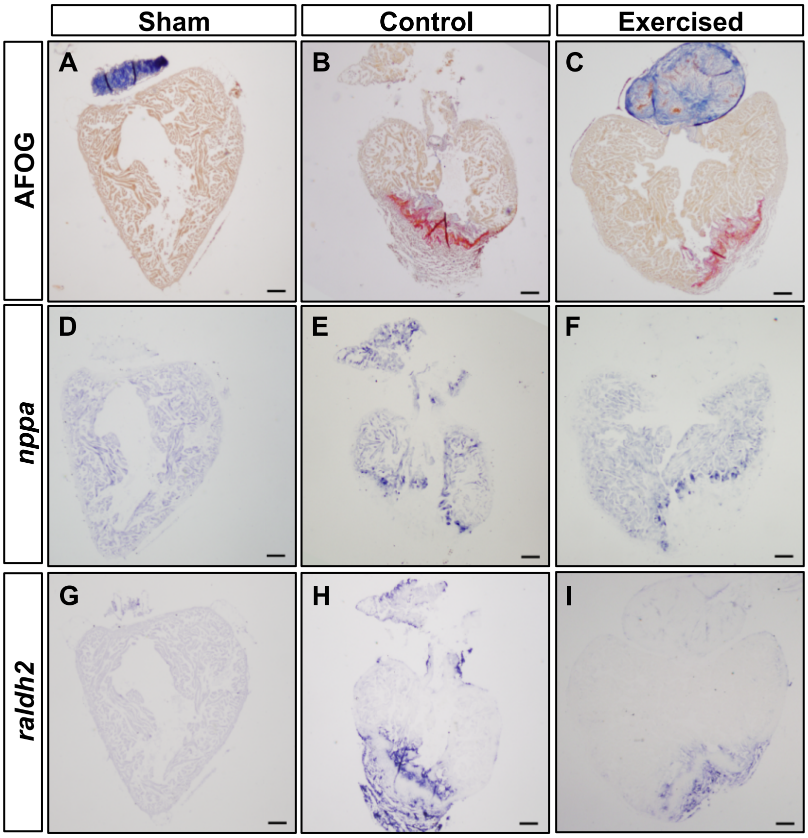

Supplementary Figure S5. Cryolesioned area at 7 dpi shown by AFOG staining and localization of mRNA expression of two known injury-response genes expressed at the injury area in hearts from injured exercised (C, F, I), non-exercised (B, E, H) and sham zebrafish (A, D, G). (A-C) AFOG staining of consecutive sections of the same hearts shown in D-I. (**D-F)** In situ hybridization of *nppa*. **(G-I)** In situ hybridization of *raldh2*. Scales bar represent 100 µm. *nppa*, atrial natriuretic peptide ; *raldh2*, retinaldehyde dehydrogenase 2.

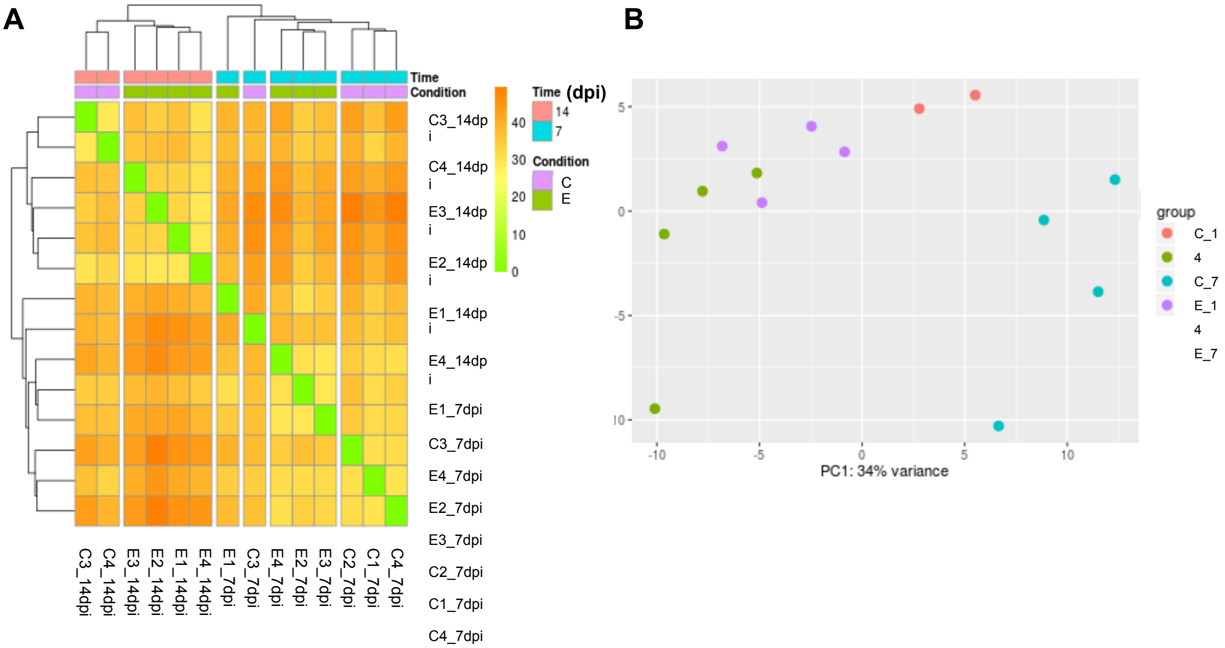

Supplementary Figure S6. Transcriptomic analysis of ventricles from exercised and non-exercised zebrafish: sample distances and distribution. (A) Sample distance matrix shows that samples are correctly clustered by time (7 and 14 dpi) and condition (exercised (E) or non-exercised (C)). Colors represent lower (green) to higher (orange) distance between samples. Sample condition and time are shown as annotation columns over the sample distance matrix. **(B)** Sample dependent principal component 1 (PC1) of log2 transformed expression values is plotted against PC2, x and y axis, respectively. Sample groups are represented by colors. In the PC analysis (PCA), PC1 reflects the effect of time, grouping 14 dpi to the right and 7 dpi to the left. dpi, days post-injury.

A. mTOR-phospho and p70S6K-phospho

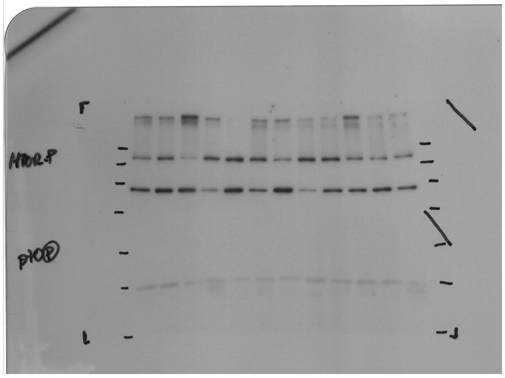

B. mTOR-total and p70S6K-total

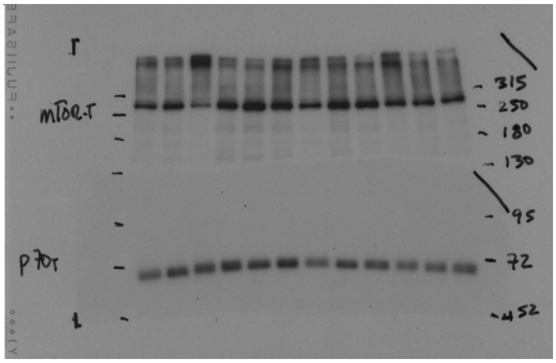

C. 4-EBP-1 phospho

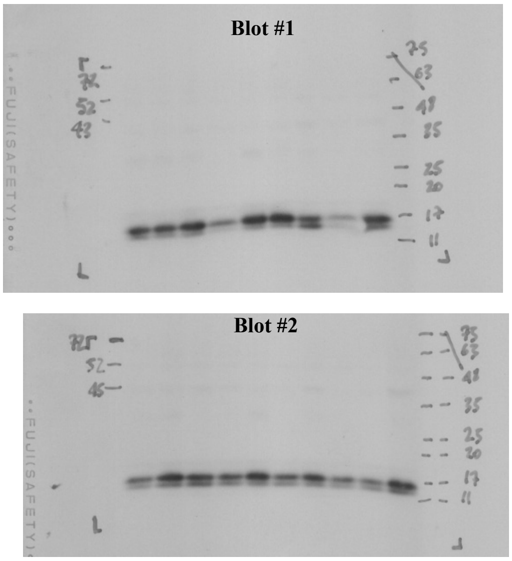

D. 4-EBP-1 total

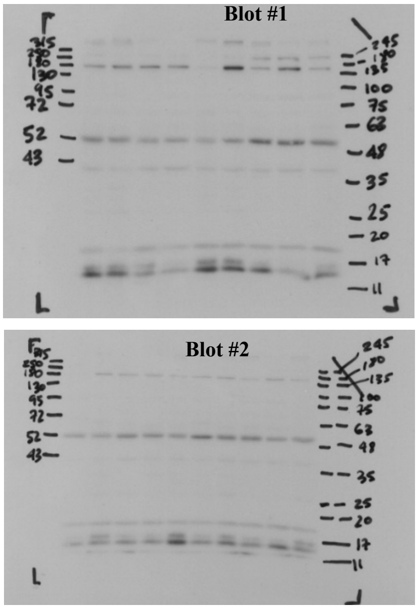

E. MEF2

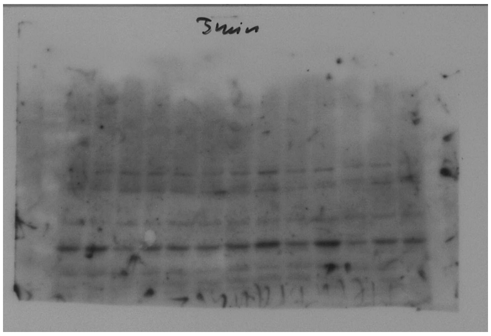

F. Tubulin

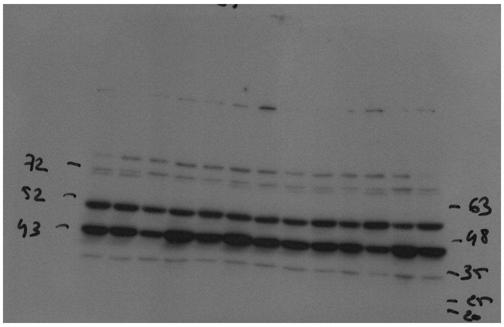

Supplementary Figure S7. Full-length blots of cropped blots shown in Figure S1. (**A**) mTOR-phospho (top) and p70S6K-phospho (bottom). (**B**) mTOR-total (top) and p70S6K-total (bottom). (**C**) 4-EBP-1-phospho: blot #1 (top), blot # 2 (bottom). (**D**) 4-EBP-1-total: blot # 1 (top), blot # 2 (bottom). **(E)** MEF2. **(F)** Tubulin.

A. p38-MAPK-phospho **B. p38-MAPK-total**

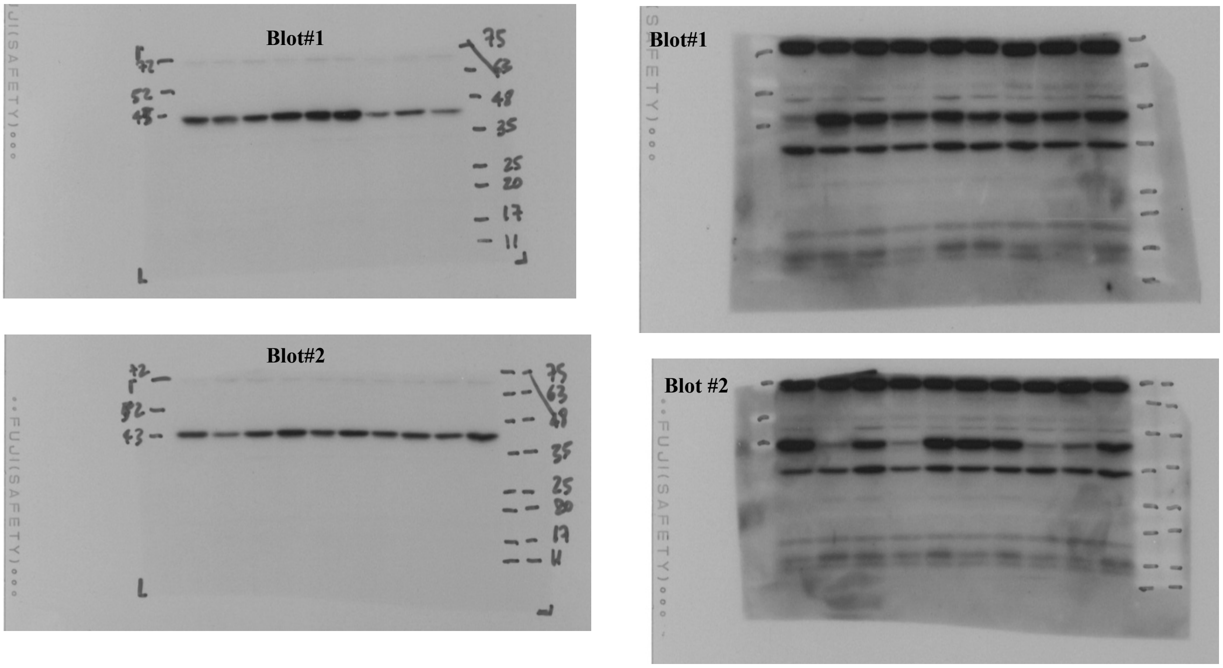

Supplementary Figure S8. Full-length blots of cropped blots shown in Figure 1. (**A**) p38-MAPK-phospho: blot #1 (top), blot # 2 (bottom). (**B**) p38-MAPK-total: blot # 1 (top), blot # 2 (bottom).
