# Supplementary material for: Physiological Responses to Swimming-Induced Exercise in the Adult Zebrafish Regenerating Heart
Source: Front Physiol. 2018 Oct 1;9:1362. doi: 10.3389/fphys.2018.01362 (PMC6174316; doi:10.3389/fphys.2018.01362)
Supplement: Supplementary file 6 [file Data_Sheet_2.docx]

**SUPPLEMENTARY METHODS**

**Sustained swimming performance test (U_crit_)**

Swimming performance was assessed in three different experimental groups: 8 dpi exercised injured fish (E; 2.78 ± 0.05 cm BL), 8 dpi non-exercised injured fish (C; 2.70 ± 0.05 cm BL) and non-injured fish (NI; 2.94 ± 0.03 cm BL). Exercised fish were induced to swim during 5 days (from 3 dpi to 7 dpi) at their optimal swimming speed (U_opt_) (Palstra et al., 2010), non-exercised fish were kept in the swim tunel at resting conditions and allowed to regenerate for 5 days; and non-injured fish were kept in the swim tunel at resting conditions for 5 days. The latter group of fish was included as a control reference for the swimming test. The swim test was assessed the next day and fish were fed 4 hours before the test. Critical swimming speed (U_crit_) was determined group-wise and the swim tunnel lid was kept loose in order to remove individual fish easily after fatigue. We considered that this modification may alter flow conditions and, consequently, the obtained U_crit_ absolute values may differ from the real U_crit_ value but since experimental conditions were the same for the three experimental group of fish, we examined the relative changes in U_crit_ between the experimental groups. A ramp-U_crit_ test was performed following previous published studies (Tierney, 2011). Speed was increased 1.6 BL/s every 5 min during the initial seven steps and every 10 min during the following steps until fish fatigued (**Supplementary Figure S4A**). U_crit_ was calculated as previously described and expressed in body lengths per second (BL/s) (Palstra et al., 2010).

**Quantitative real-time PCR**

Total RNA from exercised and non-exercised ventricles after four experimental weeks was obtained from pools of two ventricles, isolated and homogenized in 150 µl of QIAzolLysis Reagent processed with the miRNeasy MicroKit (Qiagen). One microgram of RNA or 500 ng of RNA was used for four experimental week ventricles or validation of RNA-sequencing data using the same samples than in the analysis, respectively. RNA was treated with DNAse I Amplification Grade (Life Technologies) to remove any contaminating genomic DNA and reverse transcribed using the Transcriptor First Strand cDNA Synthesis Kit (Roche) as specified by the manufacturer. Reactions were run in a CFX384™ Real-Time System (Bio-Rad) under the following thermal cycling conditions: 3 min at 95 °C and 40 cycles of 10 s at 95 °C, 30 s at the corresponding annealing temperature and a final melting curve of 81 cycles from 55 to 95 °C (0.5 °C increase every 30 s) to analyze the specificity of the reaction and absence of primer dimers. The reactions (5 µl) contained 2.5 μL of iQ SYBR Green Supermix (Bio-Rad), 500 nM of forward and reverse primers and 1 μL of cDNA for each sample (diluted 1:5) and followed the requirements of the MIQE guidelines. All PCR reactions were run in triplicate. Primer efficiency was calculated by analyzing serial dilutions of cDNA samples and was higher than 90%. The expression level of each gene was normalized to the two or three most stable reference genes tested, *rps15*, *rps18*, *rpl11* and *bactin*. (M-value < 0.5) and quantified using CFX384 software (modified (Pfaffl, 2001)). Primer sequences were designed using PerlPrimer Software or Roche Universal Probe Library Assay Design Center. Primer sequences, amplicon sizes and accession numbers are listed in **Supplementary Table S3.**

**In situ hybridization**

In situ hybridization was performed according to published studies (González-Rosa et al., 2014). Briefly, paraffin slides were dewaxed in xylene and rehydrated by a series of graded alcohols, permeabilized with proteinase K (10 µg/ml), fixed in 4%PFA/PBS and acetylated with 0.1M triethanolamine (pH8) and 0.25% acetic anhydride. After blocking for at least 2h in hybridization buffer, slides were hybridized overnight at 65ºC with hybridization buffer containing the probe. Slides were washed with hybridization buffer, MABT1X, and blocked for 2h at room temperature with blocking solution (10% blocking reagent, Roche with 20% sheep serum). Anti-DIG AP antibody (Roche) was incubated at 4ºC overnight and developed using BM-purple substrate (Roche). Slides were fixed in 4%PFA/PBS, dehydrated and mounted in DPX (Sharlau). Digoxigenin-labeled antisense RNA probes were synthesized with T7 or SP6 RNA polymerase (Ambion) after plasmid linearization. The cRNA products were precipitated with 4M LiCl and resuspended in DEPC water. RNA probes were quantified using Nanodrop2000 (Thermo Scientific) and integrity was confirmed by agarose gel electrophoresis.

**References**

González-Rosa, J. M., Guzmán-Martínez, G., Marques, I. J., Sánchez-Iranzo, H., Jiménez-Borreguero, L. J., and Mercader, N. (2014). Use of echocardiography reveals reestablishment of ventricular pumping efficiency and partial ventricular wall motion recovery upon ventricular cryoinjury in the zebrafish. *PLoS One* 9, e115604. doi:10.1371/journal.pone.0115604.

Palstra, A. P., Tudorache, C., Rovira, M., Brittijn, S. A., Burgerhout, E., van den Thillart, G. E. E. J. M., et al. (2010). Establishing Zebrafish as a Novel Exercise Model: Swimming Economy, Swimming-Enhanced Growth and Muscle Growth Marker Gene Expression. *PLoS One* 5, e14483. doi:10.1371/journal.pone.0014483.

Pfaffl, M. W. (2001). A new mathematical model for relative quantification in real-time RT-PCR. *Nucleic Acids Res.* 29, e45. Available at: http://www.pubmedcentral.nih.gov/articlerender.fcgi?artid=55695&tool=pmcentrez&rendertype=abstract.

Tierney, K. B. (2011). Swimming performance assessment in fishes. *J. Vis. Exp.* doi:10.3791/2572.
